# Construction of nomograms for predicting overall survival and progression-free survival in patients with high-grade serous ovarian carcinoma: a retrospective study

**DOI:** 10.7717/peerj.21190

**Published:** 2026-04-30

**Authors:** Yuping Shan, Kejuan Song, Zhengyi Shan, Fangling Han, Lijun Li, Huijun Chu

**Affiliations:** 1 Department of Obstetrics and Gynecology, The Affiliated Hospital of Qingdao University, Qingdao, China; 2 Department of Pathology, The Affiliated Hospital of Qingdao University, Qingdao, China; 3 Xuejiadao Community Health Service Center of the Western Coast New Area, Qingdao, China; 4 Department of Pharmacy, Qingdao Hiser Hospital, Qingdao, China

**Keywords:** High-grade serous ovarian carcinoma, Prognosis, Progression-free survival, Nomograms, Overall survival

## Abstract

**Background:**

The aim of this study was to identify independent prognostic factors and develop predictive nomograms for overall survival (OS) and progression-free survival (PFS) in patients with high-grade serous ovarian carcinoma (HGSOC).

**Methods:**

Information on patients primarily diagnosed with HGSOC at the Affiliated Hospital of Qingdao University from June 2008 to June 2018 was extracted. Kaplan–Meier (K-M) analyses were used to generate survival curves. We employed univariate and multivariate Cox regression analyses to determine independent prognostic factors, and prognostic nomograms for OS and PFS were developed.

**Results:**

A total of 573 patients were included in the final study. The age at diagnosis, first-visit interval, peripheral blood neutrophil-to-lymphocyte ratio, the immunohistochemical expressions of estrogen receptor and progesterone receptor, and Federation of Gynecology and Obstetrics (FIGO) stage were independent prognostic factors associated with OS and PFS. Additionally, the immunohistochemical expression of Wilms’ tumor-1 (WT-1) and neoadjuvant chemotherapy were also related to the OS, whereas the serum carbohydrate antigen 125 (CA125) level, the immunohistochemical expression of CK7, omentum metastasis, and postoperative adjuvant chemotherapy were independent prognostic factors linked to PFS. The area under the time-dependent receiver operating characteristic curve values of the nomograms were higher than those of the FIGO staging system. The calibration curves and decision curve analysis demonstrated the clinical applicability of the nomograms.

**Conclusion:**

We developed two new risk stratifications based on the total points of the nomograms. This study could provide a foundation for the development of more accurate predictive models that can assist clinicians in creating individualized treatment plans and improving the prognosis of HGSOC.

## Introduction

Ovarian cancer (OC) is one of the deadliest gynecological malignancies in women worldwide, with more than 313,000 cases and 207,000 deaths annually (Konstantinopoulos & Matulonis, 2023). However, there are currently no effective instruments for screening the general population, and this has economic implications. Over the past decade, researchers have explored cost-effective methods for the early detection and prevention of OC. However, the cost of treatment per patient with OC remains the highest among all cancer types (Ghose et al., 2022). For example, the average initial cost in the first year can be approximately USD 80,000, while the cost in the final year can increase to USD 100,000 (Ghose et al., 2022; Mariotto et al., 2011). Epithelial ovarian cancer (EOC) is the most common histological type of OC, accounting for almost 90% of OC cases (Liao et al., 2018). High-grade serous ovarian carcinoma (HGSOC) is the most common and lethal type of OC. The Federation of Gynecology and Obstetrics (FIGO) classification system is used to stage these tumors and plays a critical role in determining prognosis and guiding treatment options. HGSOC is commonly associated with mutations in the *TP53* gene, while *BRCA* mutations are found in approximately 15% of cases (Pavlidis et al., 2021). Owing to the lack of specific symptoms, most patients with HGSOC are diagnosed at advanced stages, and the 5-year survival rate is <40% (Punzón-Jiménez et al., 2022). Moreover, the survival rate of women with HGSOC remains low despite recent treatment advancements and significant progress in our comprehension of the molecular etiology and clinical pathology (Berek et al., 2021; Punzón-Jiménez et al., 2022; Qian et al., 2023). Therefore, it is important to define predictive factors that affect survival. This will assist doctors in making individualized prognostic assessments and treatment plans, ultimately reducing the mortality rate of patients with HGSOC.

Nomograms, as comprehensive and readable predictive models, have been utilized in recent years to assist surgeons in formulating treatment plans and assessing the prognosis of patients diagnosed with various cancer types, including lung, colorectal, cervical, endometrial, and other tumors (Cheng et al., 2023; Liu et al., 2023; Miao et al., 2023; Yang et al., 2022; Yu & Zhang, 2020). A nomogram converts complex mathematical regression models into visual graphs that intuitively convey the influence of predictive indicators by assigning scores to each factor based on their degree of influence on the outcome variables in the model (Wang et al., 2021). Compared to using only FIGO and the American Joint Committee on Cancer (AJCC) staging systems, a nomogram offers a more individualized survival prediction for each patient (Cheng et al., 2023).

To date, only a few nomograms have been validated for patients with HGSOC, but most rely on relatively limited and variable clinical data and a comparison with the FIGO staging system, clinical benefit evaluation, and risk stratification are lacking (Huang et al., 2022; Li et al., 2023). Therefore, this study was undertaken to establish and validate novel nomograms for overall survival (OS) and progression-free survival (PFS) in patients with HGSOC based on significant prognostic factors derived from the Affiliated Hospital of Qingdao University. We further compared methods for predicting prognosis using a nomogram and a separate FIGO staging system. Additionally, we used decision curve analysis (DCA) curves to assess the clinical utility of the nomograms and stratified the risk of HGSOC based on the nomograms. The goal of this study was to construct nomograms to predict the prognosis of patients with HGSOC and to promote the development of more comprehensive and reliable nomograms that can enhance individualized prognostic prediction and the treatment of HGSOC.

## Materials and Methods

### Study population selection

This study was reviewed and approved by the ethics committee of the Affiliated Hospital of Qingdao University with the approval number: QYFYWZLL 28256, dated 11/29/2023. All patients (or their proxies/legal guardians) provided written informed consent to participate in the study and for their data to be published. Informed consents were acquired from the patients when they arrived the study hospital for treatment during 2008–2018. All procedures performed in studies involving human participants were conducted in compliance with the ethical standards of the institutional and/or national research committee and in accordance with the 1964 Helsinki Declaration and its subsequent amendments or equivalent ethical standards.

This study included patients with HGSOC treated at the Affiliated Hospital of Qingdao University between June 2008 and June 2018. The inclusion criteria were as follows: (1) initial treatment at the Affiliated Hospital of Qingdao University with either chemotherapy or surgery; (2) the patient underwent surgical treatment at the Affiliated Hospital of Qingdao University; (3) diagnosis of HGSOC by the Affiliated Hospital of Qingdao University after a postoperative pathological evaluation; and (4) complete clinical data. The exclusion criteria were as follows: (1) patients with other malignant tumors, such as cervical, breast, and gastric cancer, and (2) patients with diseases that seriously affect survival, such as severe acute myocardial infarction and intracerebral hemorrhage.

### Data elements

The following variables were selected: age of diagnosis; body mass index (BMI); ABO blood group; age at menarche; age at menopause; number of pregnancies; number of miscarriages; first-visit interval; serum carbohydrate antigen 125 (CA125) level; serum human epididymis protein 4 (HE4) level; serum carcinoembryonic antigen (CEA) level; red blood cell distribution width-coefficient of variation (RDW-CV); mean corpuscular volume (MCV); mean corpuscular hemoglobin (MCH); peripheral blood neutrophil-to-lymphocyte ratio (NLR); systemic immunoinflammatory index (SII); lactate dehydrogenase (LDH); triglyceride-to-high density lipoprotein cholesterol ratio (TG/HDL-C); tumor size; tumor laterality; the immunohistochemical expression of Ki-67, CA125, CK7, P16, P53, estrogen receptor (ER), progesterone receptor (PR), PAX-8, vimentin, and Wilms’ tumor-1 (WT-1); surgical modality; R0 resection or not; intraoperative ascites or not; lymph node metastasis or not; omentum metastasis or not; FIGO stage; neoadjuvant chemotherapy (NACT) or not; and postoperative adjuvant chemotherapy or not. In particular, the SII is an indicator of peripheral blood, calculated by multiplying the platelet count by the neutrophil count and then dividing by the lymphocyte count. Except for the immunohistochemical expression, FIGO stage, and treatment information, all other data were collected before the initial treatment, and peripheral blood indices were collected for the first time prior to the initial treatment. R0 resection was defined as no visible residual focus after R0 cytoreductive surgery (CRS) (Wang et al., 2019). It should be noted that P53 immunohistochemistry was used as a surrogate marker of *TP53* status, rather than a direct representation of the underlying mutation type. Consistent with previous reports, *TP53*-mutated tumors may exhibit either complete loss of P53 staining (−), typically associated with loss-of-function variants, or strong diffuse nuclear overexpression (+++), often associated with dominant-negative variants. Intermediate staining patterns (+/++) may reflect heterogeneous expression and were therefore interpreted cautiously. Accordingly, P53 IHC categories were used for prognostic modeling purposes, while recognizing the biological heterogeneity underlying different *TP53* mutation types.

All patients were followed up every 2–4 months in the first 2 years after completing primary treatment. The follow-up interval was then extended to every 3–6 months in the subsequent 3 years and further extended to every 6–12 months thereafter. The follow-up period was until June 2023. OS and PFS were the primary study endpoints; in addition, the 3-year OS, 5-year OS, 3-year PFS, and 5-year PFS were the other outcomes of interest in our study.

### Statistical analyses and prognostic nomograms

X-tile software (Yale University, New Haven, CT, USA) was used to convert continuous variables into categorical variables by calculating the optimal cutoff points for each variable. We then utilized Kaplan–Meier (K–M) survival analyses to calculate the survival probabilities, in terms of OS and PFS, of the clinical factors and created K–M curves. Finally, log-rank tests were used to compare the differences between the curves.

To assess several prognostic variables related to OS and PFS, univariate and multivariate Cox proportional hazard regression models were used to determine hazard ratios and 95% confidence intervals. Some variables that were critical for understanding the prognosis of HGSOC were retained in the analysis despite having fewer than 10 events per level. These variables were carefully selected based on their clinical relevance and their established significance in the literature as potential prognostic factors for OS and PFS. Subsequently, two novel nomograms associated with OS and PFS were developed for patients with HGSOC. Notably, variables included in the final models were selected based on a combination of statistical results, clinical relevance, and prior evidence. Although some covariates did not reach statistical significance in the multivariable analysis, they were retained because of their established prognostic importance in HGSOC and their contribution to overall model performance. As the primary aim of this study was prognostic prediction rather than causal inference, inclusion of clinically meaningful variables was prioritized to improve model stability and interpretability.

In these analyses, (1) events were delineated by the occurrence of primary endpoints, where for OS, events were identified by death from any cause or the date on which the individual was last confirmed to be alive, and for PFS, events were indicated by disease recurrence, progression, or the date of the most recent follow-up. (2) OS was defined as the time from diagnosis to death from any cause, or last known alive; and (3) PFS was defined as the time from diagnosis to the first occurrence of disease recurrence or progression, or date of last available follow-up.

To assess the discriminatory ability of the nomograms, we used the area under the time-dependent receiver operating characteristic (ROC) curve (AUC). In addition, the performance of our nomogram was compared with that of the FIGO staging system using ROC curves. Calibration plots were created after 100 sampling repetitions, using the bootstrap method for internal validation. DCA curves were used to test the clinical applicability of the predictive models. Finally, two new risk stratification systems were developed using X-tile software based on the total points of the nomograms. Survival differences among the different risk stratification groups were compared using log-rank tests and K–M curves.

All statistical analyses were performed using SPSS software (version 29.0; IBM Corp., Armonk, NY, USA) and R software (version 4.3.2; R Core Team, 2023). A *p*-value < 0.05 was considered statistically significant.

## Results

**Figure 1:**
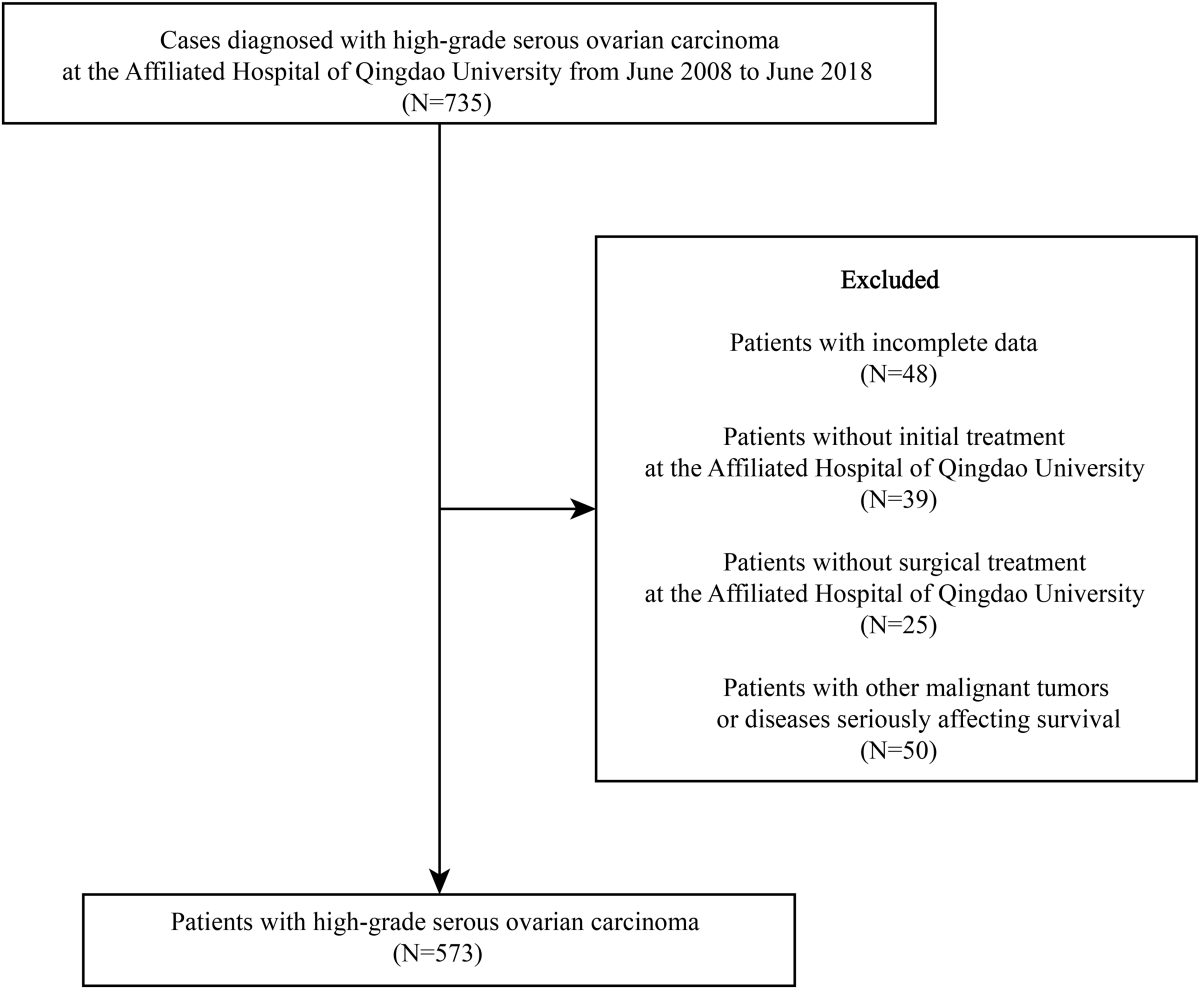
Flow diagram based on the inclusion and exclusion criteria.

In total, 735 patients diagnosed with HGSOC were identified at the Affiliated Hospital of Qingdao University between June 2008 and June 2018. Of these, 573 were included based on the inclusion and exclusion criteria (Fig. 1).

### Patient characteristics and survival outcomes

The clinical characteristics of patients are presented in Table 1. Most cases were patients under 59 years of age (63.5%), and the highest proportion of first-visit intervals was 18–120 days (60.7%). The serum CA125 level for the majority of the patients was 217.8–2,496 U/mL (61.4%), and their NLR was under 4.13 (69.3%). Although there were a considerable number of patients with omentum metastasis (61.4%) and stage IIIC disease (58.1%), most patients achieved R0 resection (82.5%) through laparotomy (87.8%). The number of NACT cycles was 2 (1, 3). Subsequently, the majority of patients underwent postoperative adjuvant chemotherapy (77.7%). The 3- and 5-year OS and PFS rates of all patients in terms of different clinical features are shown in Table 2. The median follow-up time for all patients was 49.1 months, calculated using the reverse Kaplan-Meier method.

**Table 1:** Characteristics of the 482 patients with high-grade serous ovarian carcinoma.

Characteristics	Total (*N* = 573)	
	Number	Percent
Demographic features		
Age of diagnosis (years)		
≤59	364	63.5
59–66	135	23.6
>66	74	12.9
BMI (kg/m^2^)		
≤24.46	187	32.6
24.46–26.37	226	39.4
>26.37	160	27.9
ABO blood group		
A	211	36.8
B	178	31.1
O	127	22.2
AB	57	9.9
Age at menarche (years)		
≤15	278	48.5
15–17	178	31.1
>17	117	20.4
Age at menopause (years)		
≤39	159	27.7
39–52	306	53.4
>52	108	18.8
Number of pregnancies (times)		
≤1	269	46.9
1–2	200	34.9
>2	104	18.2
Number of miscarriages (times)		
0	245	42.8
0–2	267	46.6
>2	61	10.6
General clinical features		
First visit interval (days)		
≤18	130	22.7
18–120	348	60.7
>120	95	16.6
Serum CA125 level (U/mL)		
≤217.8	116	20.2
217.8–2,496	352	61.4
2,496–5,000	69	12.0
>5,000	36	6.3
Serum HE4 level (pmol/L)		
≤668.24	216	37.7
668.24–997.8	169	29.5
997.8–1,500	112	19.5
>1,500	76	13.3
Serum CEA level (ng/mL)		
≤0.2	58	10.1
0.2–0.4	87	15.2
0.4–1.68	252	44.0
>1.68	176	30.7
RDW-CV (%)		
≤12	73	12.7
12–14.2	430	75.0
>14.2	70	12.2
MCV (fL)		
≤84.7	170	29.7
84.7–90.7	280	48.9
>90.7	123	21.5
MCH (pg)		
≤28.5	301	52.5
28.5–30.5	204	35.6
>30.5	68	11.9
NLR		
≤4.13	397	69.3
4.13–6.11	117	20.4
>6.11	59	10.3
SII		
≤1,383.95	289	50.4
1,383.95–2,384.04	229	40.0
>2,384.04	55	9.6
LDH (U/L)		
≤152.5	102	17.8
152.5–246	285	49.7
>246	186	32.5
TG/HDL-C		
≤0.5	110	19.2
0.5–0.82	143	25.0
>0.82	320	55.8
Surgical features		
Tumor size (cm)		
≤4.0	250	43.6
4.0–8.5	192	33.5
>8.5	131	22.9
Laterality		
Unilateral	242	42.2
Bilateral	331	57.8
Surgical modality		
Laparotomy	503	87.8
Laparoscopy	70	12.2
R0 resection		
No	100	17.6
Yes	473	82.5
Intraoperative ascites		
No	265	46.2
Yes	308	53.8
Lymph node metastasis		
No	419	73.1
Yes	154	26.9
Omentum metastasis		
No	221	38.6
Yes	352	61.4
FIGO stage		
IA	18	3.1
IB	13	2.3
IC	30	5.2
IIA	18	3.1
IIB	32	5.6
IIC	22	3.8
IIIA	14	2.4
IIIB	21	3.7
IIIC	333	58.1
IVA	30	5.2
IVB	42	7.3
NACT (times)		
0	298	52.0
2 (1, 3)	275	48.0
Postoperative adjuvant chemotherapy		
No	128	22.3
Yes	445	77.7
Histological features		
Ki-67 (%)		
≤50	236	41.2
50–65	195	34.0
>65	142	24.8
CA125		
–	82	14.3
+	241	42.1
++	153	26.7
+++	97	16.9
CK7		
–	118	20.6
+	310	54.1
++	125	21.8
+++	20	3.5
P16		
–	103	18.0
+	212	37.0
++	149	26.0
+++	109	19.0
P53		
–	144	25.1
+	212	37.0
++	102	17.8
+++	115	20.1
ER		
–	92	16.1
+	220	38.4
++	105	18.3
+++	156	27.2
PR		
–	165	28.8
+	129	22.5
++	156	27.2
+++	123	21.5
PAX-8		
–	117	20.4
+	280	48.9
+++	176	30.7
Vimentin		
–	261	45.5
+	93	16.2
++	219	38.2
WT-1		
–	81	14.1
+	327	57.1
++	94	16.4
+++	71	12.4

**Table 2:** The 3-and 5-year overall survival and progression-free survival in terms of patient characteristics.

Characteristics	Progression-free survival		Overall survival	
	3-year (%)	5-year (%)	3-year (%)	5-year (%)
Demographic features				
Age of diagnosis (years)				
≤59	16.8 (61/364)	2.2 (8/364)	90.4 (329/364)	22.0 (80/364)
59–66	0.0 (0/135)	0.0 (0/135)	13.3 (18/135)	0.0 (0/135)
>66	0.0 (0/74)	0.0 (0/74)	0.0 (0/74)	0.0 (0/74)
BMI (kg/m^2^)				
≤24.46	10.2 (19/187)	0.5 (1/187)	66.3 (124/187)	16.0 (30/187)
24.46–26.37	11.5 (26/226)	0.4 (1/226)	64.2 (145/226)	14.2 (32/226)
>26.37	10.0 (16/160)	3.8 (6/160)	48.8 (78/160)	11.3 (18/160)
ABO blood group				
A	9.5 (20/211)	0.5 (1/211)	56.9 (120/211)	13.3 (28/211)
B	11.2 (20/178)	1.1 (2/178)	61.8 (110/178)	14.6 (26/178)
O	8.7 (11/127)	0.8 (1/127)	59.1 (75/127)	13.4 (17/127)
AB	17.5 (10/57)	7.0 (4/57)	73.7 (42/57)	15.8 (9/57)
Age at menarche (years)				
≤15	9.7 (27/278)	0.4 (1/278)	65.5 (182/278)	12.2 (34/278)
15–17	11.8 (21/178)	2.8 (5/178)	59.0 (105/178)	16.3 (29/178)
>17	11.1 (13/117)	1.7 (2/117)	51.3 (60/117)	14.5 (17/117)
Age at menopause (years)				
≤39	8.8 (14/159)	0.6 (1/159)	79.9 (127/159)	10.1 (16/159)
39–52	10.5 (32/306)	1.3 (4/306)	56.2 (172/306)	15.4 (47/306)
>52	13.9 (15/108)	2.8 (3/108)	44.4 (48/108)	15.7 (17/108)
Number of pregnancies (times)				
≤1	11.9 (32/269)	0.7 (2/269)	76.2 (205/269)	11.2 (30/269)
1–2	8.0 (16/200)	1.0 (2/200)	57.5 (115/200)	14.5 (29/200)
>2	12.5 (13/104)	3.8 (4/104)	26.0 (27/104)	20.2 (21/104)
Number of miscarriages (times)				
0	8.6 (21/245)	2.4 (6/245)	53.5 (131/245)	13.9 (34/245)
0–2	10.1 (27/267)	0.7 (2/267)	62.9 (168/267)	12.7 (34/267)
>2	21.3 (13/61)	0.0 (0/61)	78.7 (48/61)	19.7 (12/61)
General clinical features				
First visit interval (days)				
≤18	13.8 (18/130)	3.8 (5/130)	67.7 (88/130)	20.0 (26/130)
18–120	10.9 (38/348)	0.9 (3/348)	55.7 (194/348)	13.5 (47/348)
>120	5.3 (5/95)	0.0 (0/95)	68.4 (65/95)	7.4 (7/95)
Serum CA125 level (U/mL)				
≤217.8	17.2 (20/116)	4.3 (5/116)	67.2 (78/116)	11.2 (13/116)
217.8–2,496	11.1 (39/352)	0.9 (3/352)	61.1 (215/352)	9.4 (33/352)
2,496–5,000	2.9 (2/69)	0.0 (0/69)	63.8 (44/69)	11.6 (8/69)
>5,000	0.0 (0/36)	0.0 (0/36)	27.8 (10/36)	72.2 (26/36)
Serum HE4 level (pmol/L)				
≤668.24	12.5 (27/216)	0.9 (2/216)	79.2 (171/216)	12.0 (26/216)
668.24–997.8	9.5 (16/169)	1.2 (2/169)	53.3 (90/169)	15.4 (26/169)
997.8–1,500	7.1 (8/112)	1.8 (2/112)	44.6 (50/112)	14.3 (16/112)
>1,500	13.2 (10/76)	2.6 (2/76)	47.4 (36/76)	15.8 (12/76)
Serum CEA level (ng/mL)				
≤0.2	6.9 (4/58)	0.0 (0/58)	62.1 (36/58)	8.6 (5/58)
0.2–0.4	19.5 (17/87)	5.7 (5/87)	71.3 (62/87)	23.0 (20/87)
0.4–1.68	10.3 (26/252)	1.2 (3/252)	59.5 (150/252)	12.3 (31/252)
>1.68	8.0 (14/176)	0.0 (0/176)	56.3 (99/176)	13.6 (24/176)
RDW-CV (%)				
≤12	15.1 (11/73)	2.7 (2/73)	68.5 (50/73)	15.1 (11/73)
12–14.2	10.9 (47/430)	1.2 (5/430)	66.5 (286/430)	14.4 (62/430)
>14.2	4.3 (3/70)	1.4 (1/70)	15.7 (11/70)	10.0 (7/70)
MCV (fL)				
≤84.7	5.3 (9/170)	1.8 (3/170)	61.8 (105/170)	12.4 (21/170)
84.7–90.7	12.9 (36/280)	1.8 (5/280)	57.1 (160/280)	13.6 (38/280)
>90.7	13.0 (16/123)	0.0 (0/123)	66.7 (82/123)	17.1 (21/123)
MCH (pg)				
≤28.5	8.0 (24/301)	1.7 (5/301)	63.8 (192/301)	13.3 (40/301)
28.5–30.5	12.7 (26/204)	1.0 (2/204)	66.7 (136/204)	17.6 (36/204)
>30.5	16.2 (11/68)	1.5 (1/68)	27.9 (19/68)	5.9 (4/68)
NLR				
≤4.13	15.4 (61/397)	2.0 (8/397)	75.6 (300/397)	20.2 (80/397)
4.13–6.11	0.0 (0/117)	0.0 (0/117)	6.8 (8/117)	0.0 (0/117)
>6.11	0.0 (0/59)	0.0 (0/59)	66.1 (39/59)	0.0 (0/59)
SII				
≤1,383.95	12.5 (36/289)	0.7 (2/289)	70.9 (205/289)	15.6 (45/289)
1,383.95–2,384.04	9.2 (21/229)	2.2 (5/229)	53.3 (122/229)	13.5 (31/229)
>2,384.04	7.3 (4/55)	1.8 (1/55)	36.4 (20/55)	7.3 (4/55)
LDH (U/L)				
≤152.5	11.8 (12/102)	0.0 (0/102)	77.5 (79/102)	23.5 (24/102)
152.5–246	13.0 (37/285)	1.8 (5/285)	66.7 (190/285)	15.1 (43/285)
>246	6.5 (12/186)	1.6 (3/186)	41.9 (78/186)	7.0 (13/186)
TG/HDL-C				
≤0.5	21.8 (24/110)	3.6 (4/110)	78.2 (86/110)	26.4 (29/110)
0.5–0.82	8.4 (12/143)	0.0 (0/143)	67.8 (97/143)	11.9 (17/143)
>0.82	7.8 (25/320)	1.3 (4/320)	51.3 (164/320)	10.6 (34/320)
Surgical features				
Tumor size (cm)				
≤4.0	11.6 (29/250)	2.8 (7/250)	56.4 (141/250)	16.8 (42/250)
4.0–8.5	9.4 (18/192)	0.5 (1/192)	65.6 (126/192)	10.4 (20/192)
>8.5	10.7 (14/131)	0.0 (0/131)	61.1 (80/131)	13.7 (18/131)
Laterality				
Unilateral	12.4 (30/242)	0.8 (2/242)	69.8 (169/242)	16.9 (41/242)
Bilateral	9.4 (31/331)	1.8 (6/331)	53.8 (178/331)	11.8 (39/331)
Surgical modality				
Laparotomy	11.3 (57/503)	1.6 (8/503)	63.6 (320/503)	13.1 (66/503)
Laparoscopy	5.7 (4/70)	0.0 (0/70)	38.6 (27/70)	20.0 (14/70)
R0 resection				
No	11.0 (11/100)	1.0 (1/100)	49.0 (49/100)	12.0 (12/100)
Yes	10.6 (50/473)	1.5 (7/473)	63.0 (298/473)	14.4 (68/473)
Intraoperative ascites				
No	14.3 (38/265)	1.9 (5/265)	64.2 (170/265)	19.6 (52/265)
Yes	7.5 (23/308)	1.0 (3/308)	57.5 (177/308)	9.1 (28/308)
Lymph node metastasis				
No	11.0 (46/419)	0.7 (3/419)	73.3 (307/419)	15.0 (63/419)
Yes	9.7 (15/154)	3.2 (5/154)	26.0 (40/154)	11.0 (17/154)
Omentum metastasis				
No	16.7 (37/221)	2.3 (5/221)	72.4 (160/221)	20.8 (46/221)
Yes	6.8 (24/352)	0.9 (3/352)	53.1 (187/352)	9.7 (34/352)
FIGO stage				
IA	55.6 (10/18)	33.3 (6/18)	83.3 (15/18)	61.1 (11/18)
IB	61.5 (8/13)	7.7 (1/13)	92.3 (12/13)	61.5 (8/13)
IC	83.3 (25/30)	3.3 (1/30)	90.0 (27/30)	90.0 (27/30)
IIA	5.6 (1/18)	0.0 (0/18)	77.8 (14/18)	77.8 (14/18)
IIB	9.4 (3/32)	0.0 (0/32)	87.5 (28/32)	59.4 (19/32)
IIC	9.1 (2/22)	0.0 (0/22)	90.9 (20/22)	0.0 (0/22)
IIIA	7.1 (1/14)	0.0 (0/14)	92.9 (13/14)	0.0 (0/14)
IIIB	4.8 (1/21)	0.0 (0/21)	90.5 (19/21)	0.0 (0/21)
IIIC	3.0 (10/333)	0.0 (0/333)	58.9 (196/333)	0.3 (1/333)
IVA	0.0 (0/30)	0.0 (0/30)	10.0 (3/30)	0.0 (0/30)
IVB	0.0 (0/42)	0.0 (0/42)	0.0 (0/42)	0.0 (0/42)
NACT (times)				
0	6.0 (18/298)	0.7 (2/298)	60.4 (180/298)	13.4 (40/298)
2 (1, 3)	15.6 (43/275)	2.2 (6/275)	60.7 (167/275)	14.5 (40/275)
Postoperative adjuvant chemotherapy				
No	8.6 (11/128)	3.1 (4/128)	17.2 (22/128)	13.3 (17/128)
Yes	11.2 (50/445)	0.9 (4/445)	73.0 (325/445)	14.2 (63/445)
Histological features				
Ki-67 (%)				
≤50	8.5 (20/236)	0.0 (0/236)	74.2 (175/236)	12.7 (30/236)
50–65	13.8 (27/195)	1.0 (2/195)	61.5 (120/195)	16.9 (33/195)
>65	9.9 (14/142)	4.2 (6/142)	36.6 (52/142)	12.0 (17/142)
CA125				
–	19.5 (16/82)	6.1 (5/82)	70.7 (58/82)	26.8 (22/82)
+	8.3 (20/241)	0.0 (0/241)	62.2 (150/241)	12.0 (29/241)
++	11.1 (17/153)	0.7 (1/153)	62.1 (95/153)	10.5 (16/153)
+++	8.2 (8/97)	2.1 (2/97)	45.4 (44/97)	13.4 (13/97)
CK7				
–	16.9 (20/118)	0.8 (1/118)	69.5 (82/118)	24.6 (29/118)
+	8.1 (25/310)	1.6 (5/310)	58.7 (182/310)	11.6 (36/310)
++	10.4 (13/125)	1.6 (2/125)	57.6 (72/125)	11.2 (14/125)
+++	15.0 (3/20)	0.0 (0/20)	55.0 (11/20)	5.0 (1/20)
P16				
–	19.4 (20/103)	0.0 (0/103)	68.9 (71/103)	37.9 (39/103)
+	6.1 (13/212)	0.5 (1/212)	80.2 (170/212)	13.2 (28/212)
++	9.4 (14/149)	4.0 (6/149)	61.7 (92/149)	14.1 (21/149)
+++	12.8 (14/109)	0.9 (1/109)	12.8 (14/109)	11.0 (12/109)
P53				
–	16.7 (24/144)	1.4 (2/144)	74.3 (107/144)	24.3 (35/144)
+	10.8 (23/212)	0.9 (2/212)	64.2 (136/212)	11.8 (25/212)
++	12.7 (13/102)	3.9 (4/102)	63.7 (65/102)	11.8 (12/102)
+++	0.9 (1/115)	0.0 (0/115)	33.9 (39/115)	7.0 (8/115)
ER				
–	2.2 (2/92)	1.1 (1/92)	14.1 (13/92)	7.6 (7/92)
+	5.9 (13/220)	0.9 (2/220)	56.8 (125/220)	3.6 (8/220)
++	15.2 (16/105)	1.9 (2/105)	90.5 (95/105)	14.3 (15/105)
+++	19.2 (30/156)	1.9 (3/156)	73.1 (114/156)	32.1 (50/156)
PR				
–	3.6 (6/165)	0.6 (1/165)	18.2 (30/165)	4.8 (8/165)
+	10.9 (14/129)	0.8 (1/129)	79.1 (102/129)	9.3 (12/129)
++	9.6 (15/156)	1.3 (2/156)	84.6 (132/156)	12.2 (19/156)
+++	21.1 (26/123)	3.3 (4/123)	67.5 (83/123)	33.3 (41/123)
PAX-8				
–	15.4 (18/117)	0.0 (0/117)	76.9 (90/117)	18.8 (22/117)
+	12.1 (34/280)	1.8 (5/280)	65.7 (184/280)	16.8 (47/280)
+++	5.1 (9/176)	1.7 (3/176)	41.5 (73/176)	6.3 (11/176)
Vimentin				
–	13.0 (34/261)	1.9 (5/261)	57.5 (150/261)	14.6 (38/261)
+	16.1 (15/93)	1.1 (1/93)	64.5 (60/93)	22.6 (21/93)
++	5.5 (12/219)	0.9 (2/219)	62.6 (137/219)	9.6 (21/219)
WT-1				
–	18.5 (15/81)	4.9 (4/81)	63.0 (51/81)	24.7 (20/81)
+	8.9 (29/327)	0.6 (2/327)	72.5 (237/327)	11.6 (38/327)
++	9.6 (9/94)	1.1 (1/94)	34.0 (32/94)	13.8 (13/94)
+++	11.3 (8/71)	1.4 (1/71)	38.0 (27/71)	12.7 (9/71)

### Survival analyses and prognostic factors

Figures S1 and S2 display the K–M survival curves. According to K–M survival analyses, the age at diagnosis, BMI, first-visit interval, serum CA125 level, serum HE4 level, serum CEA level, NLR, SII, TG/HDL-C, immunohistochemical expression of Ki-67, CA125, P53, ER, PR, PAX-8, and WT-1, R0 resection or not, intraoperative ascites or not, lymph node metastasis or not, omentum metastasis or not, NACT or not, postoperative adjuvant chemotherapy or not, and FIGO stage affected the OS and PFS of patients with HGSOC. Additionally, the LDH, tumor laterality, and immunohistochemical expression of P16 also affected the OS of patients with HGSOC. In addition, the immunohistochemical expression of CK7 affected patient PFS.

Univariate and multivariate Cox proportional hazard regression analyses for OS and PFS are presented in Tables 3 and 4, respectively. These analyses were performed on dichotomized variables, with continuous variables categorized using optimal cutoff points determined by statistical methods, such as X-tile software. According to the results of univariate Cox regression analyses, the age at diagnosis, BMI, first-visit interval, serum CA125 level, serum HE4 level, NLR, SII, TG/HDL-C, immunohistochemical expressions of Ki-67, CA125, P53, ER, PR, PAX-8, and WT-1, intraoperative ascites or not, lymph node metastasis or not, omentum metastasis or not, FIGO stage, NACT or not, and postoperative adjuvant chemotherapy or not were potentially correlated with the OS and PFS of patients with HGSOC. Besides, serum CEA level, LDH, tumor laterality, the immunohistochemical expression of P16, and R0 resection or not were risk factors for OS. Moreover, the immunohistochemical expression of CK7 was identified as a risk factor for PFS These potential prognostic factors were further assessed using multivariate Cox proportional hazard regression analyses, which indicated that the age at diagnosis, first-visit interval, NLR, ER, PR, WT-1, FIGO stage, and NACT were independent prognostic factors associated with the OS of HGSOC. Moreover, the age at diagnosis, first-visit interval, serum CA125 level, NLR, CK7, ER, PR, omentum metastasis, FIGO stage, and postoperative adjuvant chemotherapy were confirmed to be independent prognostic indicators of PFS. Although the number of events in the “Age of diagnosis >66” group is relatively small, age remains a key prognostic factor for OS and PFS. Older patients often present with different clinical characteristics that significantly impact their survival, such as comorbidities and lower treatment tolerance. Despite the lower event count, we decided to retain this group as it provides important prognostic information. Similarly, patients in the advanced stages (FIGO stage IVA and IVB) of HGSOC are at a high risk of poor outcomes, and we chose to keep these stages separate in the analysis. Pooling these stages might obscure potential differences in prognosis between them, and both stages are clinically significant in their own right. Despite having fewer events in these categories, their inclusion is crucial for accurately reflecting the prognosis of advanced-stage patients.

**Table 3:** Univariate and multivariable Cox proportional hazard regression analysis of overall survival.

Characteristics	Univariate analysis		Multivariate analysis	
	Hazard ratios (95% CI)	*p*-value	Hazard ratios (95% CI)	*p*-value
Demographic features				
Age of diagnosis (years)		<0.001		
≤59	Reference		Reference	
59–66	38.578 [25.899–57.635]		17.148 [9.838–29.996]	<0.001
>66	734.102 [392.648–1,368.550]		118.955 [48.827–289.856]	<0.001
BMI (kg/m^2^)		0.011		
≤24.46	Reference		Reference	
24.46–26.37	1.386 [1.283–1.567]		1.173 [0.818–1.462]	0.623
>26.37	1.598 [1.123–1.778]		1.289 [0.925–1.967]	0.151
ABO blood group		0.136		
A	Reference		–	
B	0.919 [0.765–1.134]		–	
O	0.997 [0.775–1.287]		–	
AB	0.698 [0.537–0.965]		–	
Age at menarche (years)		0.481		
≤15	Reference		–	
15–17	0.997 [0.791–1.198]		–	
>17	1.142 [0.891–1.440]		–	
Age at menopause (years)		0.433		
≤39	Reference		–	
39–52	1.117 [0.996–1.383]		–	
>52	1.189 [0.906–1.561]		–	
Number of pregnancies (times)		0.323		
≤1	Reference		–	
1–2	1.263 [0.956–1.829]		–	
>2	1.046 [0.800–1.361]		–	
Number of miscarriages (times)		0.398		
0	Reference		–	
0–2	0.952 [0.787–1.155]		–	
>2	0.817 [0.610–1.094]		–	
General clinical features				
First visit interval (days)		0.012		
≤18	Reference		Reference	
18–120	1.259 [1.100–1.536]		1.397 [1.177–1.812]	0.012
>120	1.545 [1.158–2.175]		1.823 [1.305–2.546]	<0.001
Serum CA125 level (U/mL)		0.012		
≤217.8	Reference		Reference	
217.8–2,496	1.386 [1.096–1.748]		0.972 [0.653–1.354]	0.826
2,496–5,000	1.508 [1.080–2.118]		1.095 [0.753–1.596]	0.642
>5,000	1.7443 [1.155–2.629]		1.370 [0.876–2.138]	0.167
Serum HE4 level (pmol/L)		0.006		
≤668.24	Reference		Reference	
668.24–997.8	1.368 [1.093–1.710]		0.812 [0.598–1.199]	0.177
997.8–1,500	1.433 [1.118–1.838]		1.137 [0.825–1.564]	0.437
>1,500	1.436 [1.080–1.908]		1.121 [0.791–1.607]	0.508
Serum CEA level (ng/mL)		0.022		
≤0.2	Reference		Reference	
0.2–0.4	0.605 [0.418–0.873]		0.877 [0.576–1.333]	0.536
0.4–1.68	0.864 [0.630–1.181]		0.819 [0.555–1.181]	0.282
>1.68	0.891 [0.643–1.236]		0.820 [0.555–1.188]	0.283
RDW-CV (%)		0.404		
≤12	Reference		–	
12–14.2	1.172 [0.888–1.555]		–	
>14.2	1.270 [0.887–1.826]		–	
MCV (fL)		0.441		
≤84.7	Reference		–	
84.7–90.7	0.937 [0.759–1.156]		–	
>90.7	0.847 [0.656–1.092]		–	
MCH (pg)		0.098		
≤28.5	Reference		–	
28.5–30.5	0.979 [0.792–1.163]		–	
>30.5	0.727 [0.549–0.973]		–	
NLR		<0.001		
≤4.13	Reference		Reference	
4.13–6.11	14.301 [10.771–18.983]		7.204 [4.769–10.882]	<0.001
>6.11	293.425 [175.019–491.664]		82.959 [31.972–215.224]	<0.001
SII		0.002		
≤1,383.95	Reference		Reference	
1,383.95–2,384.04	1.236 [1.021–1.496]		1.133 [0.896–1.430]	0.299
>2,384.04	1.674 [1.233–2.273]		0.942 [0.625–1.422]	0.776
LDH (U/L)		0.026		
≤152.5	Reference		Reference	
152.5–246	1.122 [0.876–1.435]		1.028 [0.769–1.374]	0.858
>246	1.395 [1.072–1.813]		1.288 [0.948–1.749]	0.106
TG/HDL-C		<0.001		
≤0.5	Reference		Reference	
0.5–0.82	1.530 [1.162–2.015]		1.199 [0.881–1.623]	0.276
>0.82	1.623 [1.270–2.051]		1.206 [0.922–1.591]	0.189
Surgical features				
Tumor size (cm)		0.791		
≤4.0	Reference		–	
4.0–8.5	1.030 [0.837–1.267]		–	
>8.5	1.086 [0.859–1.377]		–	
Laterality		0.028		
Unilateral	Reference		Reference	
Bilateral	1.229 [1.023–1.474]		0.955 [0.773–1.174]	0.646
Surgical modality		0.601		
Laparotomy	Reference		–	
Laparoscopy	0.929 [0.712–1.227]		–	
R0 resection		0.032		
No	Reference		Reference	
Yes	0.771 [0.618–0.979]		0.958 [0.722–1.275]	0.770
Intraoperative ascites		0.006		
No	Reference		Reference	
Yes	1.289 [1.077–1.545]		0.991 [0.802–1.222]	0.925
Lymph node metastasis		0.015		
No	Reference		Reference	
Yes	1.295 [1.053–1.592]		1.153 [0.916–1.466]	0.249
Omentum metastasis		<0.001		
No	Reference		Reference	
Yes	1.461 [1.209–1.760]		1.218 [0.974–1.499]	0.086
FIGO stage		<0.001		
IA	Reference		Reference	
IB	1.530 [0.672–3.479]		1.226 [0.483–3.111]	0.669
IC	2.505 [1.174–5.392]		3.421 [1.421–8.223]	0.006
IIA	5.698 [2.416–13.494]		8.129 [2.880–22.934]	<0.001
IIB	6.971 [3.170–15.326]		10.915 [4.230–28.116]	<0.001
IIC	11.933 [4.973–28.632]		19.551 [6.813–56.097]	<0.001
IIIA	18.482 [7.156–47.737]		54.470 [17.602–168.603]	<0.001
IIIB	24.395 [10.028–59.342]		101.256 [33.930–302.173]	<0.001
IIIC	92.610 [42.643–201.127]		323.333 [117.552–888.915]	<0.001
IVA	472.323 [196.791–1,133.542]		721.128 [216.134–2,406.033]	<0.001
IVB	862.487 [366.622–2,029.024]		1,600.027 [499.360–5,231.498]	<0.001
NACT (times)		0.030		
0	Reference		Reference	
2 (1, 3)	0.819 [0.684–0.982]		0.801 [0.647–0.999]	0.039
Postoperative adjuvant chemotherapy		<0.001		
No	Reference		Reference	
Yes	0.683 [0.550–0.849]		0.811 [0.624–1.059]	0.123
Histological features				
Ki-67 (%)		0.017		
≤50	Reference		Reference	
50–65	1.252 [1.014–1.554]		1.251 [0.958–1.638]	0.101
>65	1.372 [1.084–1.777]		1.070 [0.809–1.418]	0.634
CA125		0.005		
–	Reference		Reference	
+	1.418 [1.070–1.881]		1.073 [0.781–1.487]	0.664
++	1.618 [1.198–2.183]		1.345 [0.960–1.885]	0.085
+++	1.714 [1.230–2.361]		0.941 [0.653–1.378]	0.742
CK7		0.065		
–	Reference		–	
+	1.369 [1.085–1.729]		–	
++	1.309 [0.994–1.723]		–	
+++	1.166 [0.639–2.124]		–	
P16		0.020		
–	Reference		Reference	
+	1.306 [1.005–1.700]		1.123 [0.812–1.553]	0.482
++	1.421 [1.074–1.881]		1.098 [0.781–1.544]	0.591
+++	1.578 [1.170–2.131]		0.956 [0.659–1.389]	0.815
P53		0.031		
–	Reference		Reference	
+	1.296 [1.026–1.638]		0.962 [0.727–1.269]	0.779
++	1.350 [1.021–1.786]		0.993 [0.706–1.369]	0.919
+++	1.457 [1.113–1.906]		0.968 [0.707–1.326]	0.840
ER		<0.001		
–	Reference		Reference	
+	0.633 [0.481–0.833]		0.537 [0.352–0.821]	0.004
++	0.264 [0.194–0.361]		0.197 [0.122–0.317]	<0.001
+++	0.255 [0.191–0.340]		0.551 [0.367–0.826]	0.004
PR		<0.001		
–	Reference		Reference	
+	0.452 [0.349–0.584]		1.049 [0.735–1.496]	0.793
++	0.301 [0.235–0.396]		0.684 [0.475–0.986]	0.042
+++	0.267 [0.205–0.348]		0.457 [0.322–0.649]	<0.001
PAX-8		0.008		
–	Reference		Reference	
+	1.131 [0.898–1.425]		0.947 [0.727–1.232]	0.685
+++	1.462 [1.136–1.884]		1.032 [0.758–1.406]	0.841
Vimentin		0.131		
–	Reference		–	
+	0.813 [0.629–1.052]		–	
++	1.066 [0.875–1.299]		–	
WT-1		0.002		
–	Reference		Reference	
+	1.319 [1.004–1.732]		1.324 [0.957–1.836]	0.090
++	1.738 [1.252–2.413]		1.899 [1.248–2.865]	0.003
+++	1.771 [1.253–2.503]		1.192 [0.772–1.841]	0.429

**Table 4:** Univariate and multivariable Cox proportional hazard regression analysis of progression-free survival.

Characteristics	Univariate analysis		Multivariate analysis	
	Hazard ratios (95% CI)	*p*-value	Hazard ratios (95% CI)	*p*-value
Demographic features				
Age of diagnosis (years)		<0.001		
≤59	Reference		Reference	
59–66	7.345 [5.677–9.501]		3.359 [2.106–5.358]	<0.001
>66	9.501 [30.711–64.610]		6.835 [3.044–15.347]	<0.001
BMI (kg/m^2^)		0.041		
≤24.46	Reference		Reference	
24.46–26.37	1.194 [0.961–1.483]		0.789 [0.611–1.019]	0.069
>26.37	1.332 [1.063–1.669]		0.897 [0.683–1.179]	0.435
ABO blood group		0.067		
A	Reference		–	
B	0.894 [0.716–1.115]		–	
O	1.018 [0.801–1.294]		–	
AB	0.673 [0.490–0.925]		–	
Age at menarche (years)		0.204		
≤15	Reference		–	
15–17	0.976 [0.794–1.200]		–	
>17	1.210 [0.954–1.537]		–	
Age at menopause (years)		0.344		
≤39	Reference		–	
39–52	1.108 [0.899–1.366]		–	
>52	1.218 [0.931–1.593]		–	
Number of pregnancies (times)		0.267		
≤1	Reference		–	
1–2	1.166 [0.957–1.421]		–	
>2	0.996 [0.765–1.295]		–	
Number of miscarriages (times)		0.176		
0	Reference		–	
0–2	0.927 [0.765–1.123]		–	
>2	0.758 [0.566–1.015]		–	
General clinical features				
First visit interval (days)		0.003		
≤18	Reference		Reference	
18–120	1.286 [1.029–1.607]		1.515 [1.187–1.934]	<0.001
>120	1.651 [1.233–2.212]		2.471 [1.775–3.440]	<0.001
Serum CA125 level (U/mL)		0.011		
≤217.8	Reference		Reference	
217.8–2,496	1.389 [1.100–1.753]		1.040 [0.807–1.341]	0.760
2,496–5,000	1.617 [1.157–2.260]		1.388 [0.967–1.992]	0.075
>5,000	1.623 [1.077–2.446]		0.548 [0.336–0.894]	0.016
Serum HE4 level (pmol/L)		0.006		
≤668.24	Reference		Reference	
668.24–997.8	1.341 [1.074–1.675]		1.007 [0.753–1.346]	0.964
997.8–1,500	1.423 [1.106–1.831]		1.011 [0.729–1.401]	0.950
>1,500	1.469 [1.108–1.947]		1.291 [0.915–1.822]	0.145
Serum CEA level (ng/mL)		0.050		
≤0.2	Reference		–	
0.2–0.4	0.653 [0.454–0.941]		–	
0.4–1.68	0.881 [0.644–1.207]		–	
>1.68	0.942 [0.679–1.305]		–	
RDW-CV (%)		0.278		
≤12	Reference		–	
12–14.2	1.135 [0.862–1.495]		–	
>14.2	1.336 [0.935–1.911]		–	
MCV (fL)		0.255		
≤84.7	Reference		–	
84.7–90.7	0.892 [0.724–1.100]		–	
>90.7	0.809 [0.628–1.043]		–	
MCH (pg)		0.059		
≤28.5	Reference		–	
28.5–30.5	0.969 [0.799–1.175]		–	
>30.5	0.706 [0.528–0.944]		–	
NLR		<0.001		
≤4.13	Reference		Reference	
4.13–6.11	7.170 [5.524–9.307]		1.783 [1.209–2.631]	0.004
>6.11	77.174 [50.428–118.104]		4.833 [1.972–11.846]	<0.001
SII		0.004		
≤1,383.95	Reference		Reference	
1,383.95–2,384.04	1.181 [0.976–1.429]		0.984 [0.781–1.238]	0.889
>2,384.04	1.641 [1.211–2.224]		0.771 [0.502–1.184]	0.235
LDH (U/L)		0.116		
≤152.5	Reference		–	
152.5–246	1.070 [0.836–1.368]		–	
>246	1.277 [0.983–1.659]		–	
TG/HDL-C		<0.001		
≤0.5	Reference		Reference	
0.5–0.82	1.574 [1.195–2.072]		0.913 [0.665–1.255]	0.575
>0.82	1.580 [1.245–2.006]		1.243 [0.941–1.641]	0.126
Surgical features				
Tumor size (cm)		0.745		
≤4.0	Reference		–	
4.0–8.5	1.044 [0.848–1.284]		–	
>8.5	1.094 [0.867–1.382]		–	
Laterality		0.076		
Unilateral	Reference		–	
Bilateral	1.178 [0.983–1.413]		–	
Surgical modality		0.975		
Laparotomy	Reference		–	
Laparoscopy	1.005 [0.759–1.329]		–	
R0 resection		0.051		
No	Reference		–	
Yes	0.790 [0.624–1.001]		–	
Intraoperative ascites		0.008		
No	Reference		Reference	
Yes	1.276 [1.066–1.528]		1.070 [0.874–1.310]	0.513
Lymph node metastasis		0.020		
No	Reference		Reference	
Yes	1.280 [1.040–1.574]		0.930 [0.725–1.193]	0.569
Omentum metastasis		<0.001		
No	Reference		Reference	
Yes	1.430 [1.186–1.723]		1.356 [1.091–1.685]	0.006
FIGO stage		<0.001		
IA	Reference		Reference	
IB	1.697 [0.752–3.827]		1.544 [0.622–3.832]	0.349
IC	2.194 [1.074–4.484]		2.611 [1.171–5.821]	0.019
IIA	4.576 [2.009–10.426]		6.164 [2.372–16.018]	<0.001
IIB	4.990 [2.354–10.575]		6.873 [2.912–16.223]	<0.001
IIC	5.568 [2.496–12.420]		4.256 [1.642–11.031]	0.003
IIIA	8.111 [3.369–19.525]		18.714 [6.817–51.375]	<0.001
IIIB	8.378 [3.727–18.833]		12.661 [4.978–32.201]	<0.001
IIIC	21.817 [11.043–43.100]		30.068 [13.143–68.792]	<0.001
IVA	148.574 [67.102–328.966]		118.367 [42.605–328.851]	<0.001
IVB	214.409 [99.048–464.129]		97.805 [35.354–270.577]	<0.001
NACT (times)		0.011		
0	Reference		Reference	
2 (1, 3)	0.792 [0.661–0.949]		0.815 [0.662–1.004]	0.055
Postoperative adjuvant chemotherapy		<0.001		
No	Reference		Reference	
Yes	0.667 [0.538–0.828]		0.653 [0.501–0.851]	0.002
Histological features				
Ki-67 (%)		0.045		
≤50	Reference		Reference	
50–65	1.150 [0.933–1.417]		0.951 [0.739–1.223]	0.696
>65	1.343 [1.063–1.697]		1.073 [0.818–1.407]	0.611
CA125		0.013		
–	Reference		Reference	
+	1.416 [1.071–1.871]		1.177 [0.857–1.616]	0.315
++	1.514 [1.124–2.039]		1.026 [0.728–1.445]	0.883
+++	1.666 [1.207–2.300]		1.093 [0.764–1.565]	0.626
CK7		0.016		
–	Reference		Reference	
+	1.459 [1.156–1.841]		1.751 [1.335–2.296]	<0.001
++	1.374 [1.044–1.807]		1.604 [1.164–2.209]	0.004
+++	1.201 [0.673–2.143]		2.539 [1.286–5.015]	0.007
P16		0.071		
–	Reference		–	
+	1.282 [0.988–1.664]		–	
++	1.323 [1.002–1.746]		–	
+++	1.473 [1.095–1.981]		–	
P53		0.044		
–	Reference		Reference	
+	1.263 [1.001–1.593]		1.004 [0.763–1.319]	0.980
++	1.324 [1.002–1.749]		1.224 [0.890–1.684]	0.214
+++	1.438 [1.100–1.880]		1.009 [0.740–1.375]	0.957
ER		<0.001		
–	Reference		Reference	
+	0.563 [0.429–0.738]		0.481 [0.325–0.713]	<0.001
++	0.269 [0.197–0.366]		0.344 [0.222–0.533]	<0.001
+++	0.272 [0.205–0.363]		0.602 [0.408–0.887]	0.010
PR		<0.001		
–	Reference		Reference	
+	0.433 [0.335–0.560]		0.774 [0.547–1.093]	0.146
++	0.333 [0.261–0.426]		0.794 [0.564–1.117]	0.185
+++	0.283 [0.218–0.368]		0.556 [0.403–0.766]	<0.001
PAX-8		0.024		
–	Reference		Reference	
+	1.145 [0.910–1.440]		1.095 [0.839–1.430]	0.503
+++	1.408 [1.093–1.813]		0.952 [0.699–1.296]	0.753
Vimentin		0.116		
–	Reference		–	
+	0.840 [0.650–1.087]		–	
++	1.111 [0.912–1.354]		–	
WT-1		0.002		
–	Reference		Reference	
+	1.249 [0.953–1.635]		0.980 [0.718–1.337]	0.898
++	1.632 [1.178–2.259]		1.043 [0.699–1.555]	0.837
+++	1.762 [1.248–2.489]		0.909 [0.594–1.391]	0.660

### Development of nomograms

**Figure 2:**
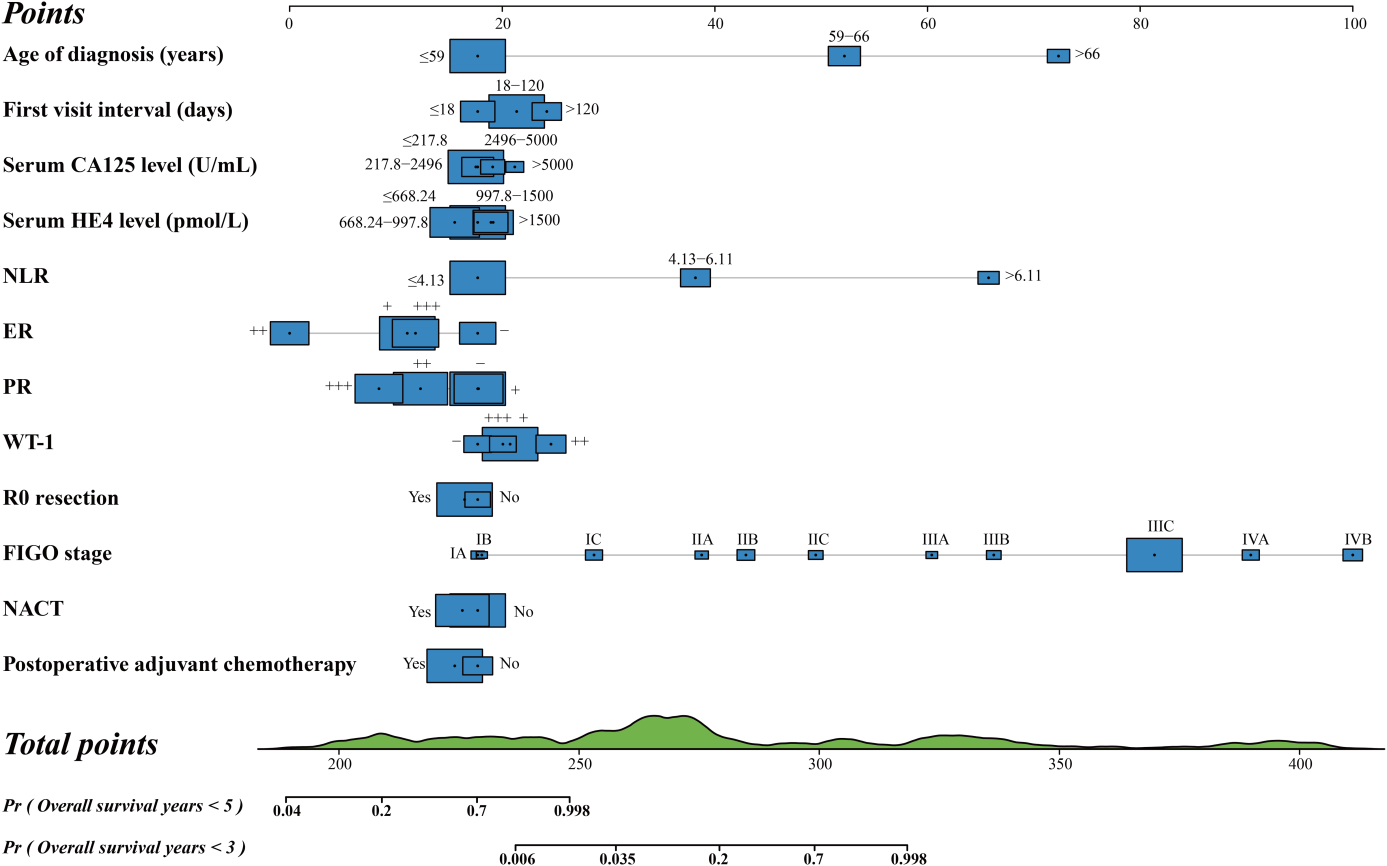
Nomogram for predicting 3- and 5-year overall survival.

**Figure 3:**
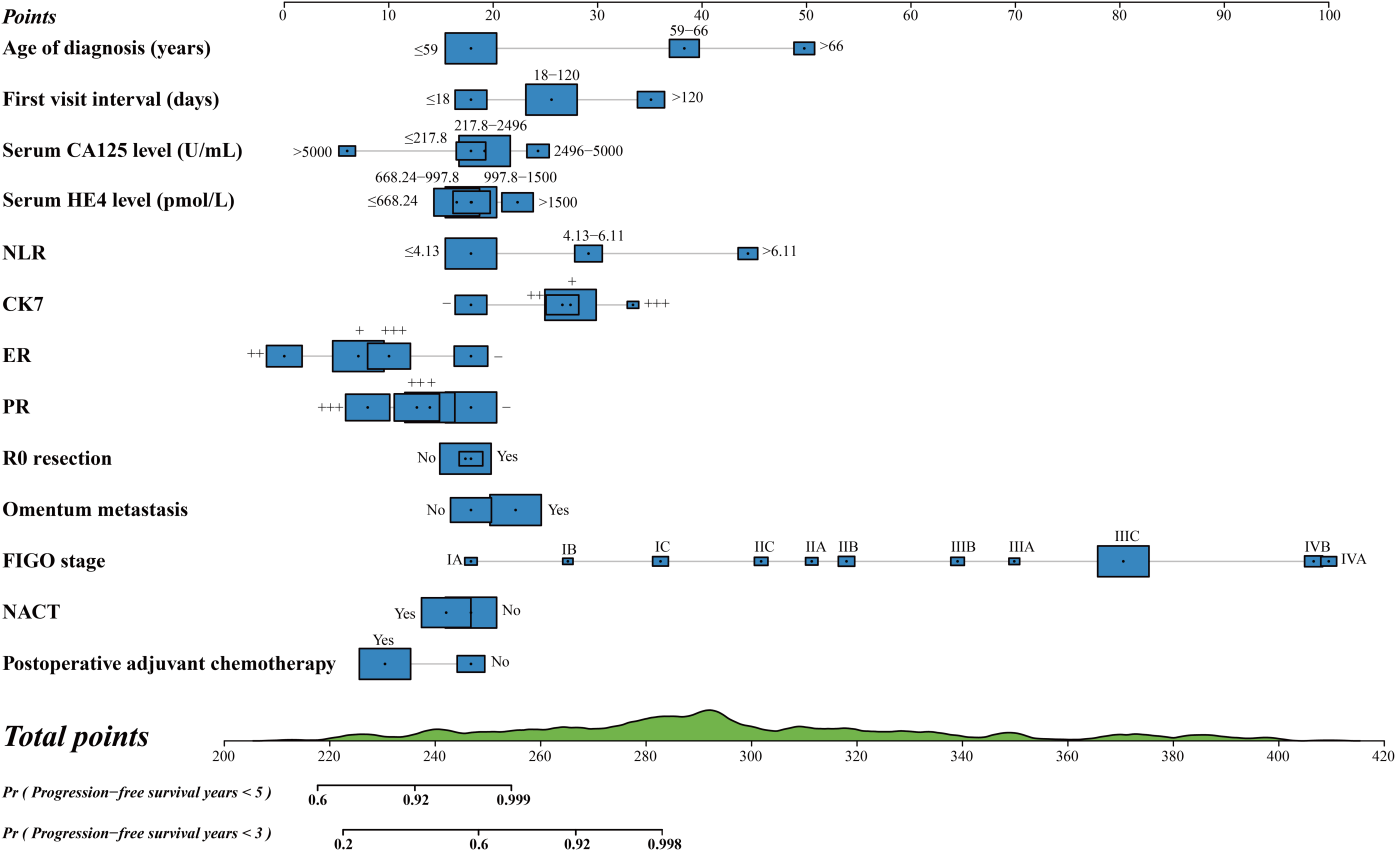
Nomogram for predicting 3 and 5-year progression-free survival.

Prognostic nomograms for predicting the OS and PFS at 3 and 5 years were constructed independently, based on the prognostic variables obtained (Figs. 2 and 3). In particular, we selected serum CA125 and HE4 levels, R0 resection, and postoperative adjuvant chemotherapy to construct a nomogram for OS, although these variables showed no significant differences in the multivariate Cox proportional hazard analyses of OS. Similarly, serum HE4 levels, R0 resection, and NACT were used to create a nomogram for the PFS of patients with HGSOC. Although these variables showed no significant differences in the multivariate Cox proportional hazard analyses of OS, they were included in the model due to their established clinical relevance in the prognosis of HGSOC, as reported in previous studies (Bates et al., 2024). Their inclusion was intended to ensure that all potentially relevant prognostic factors were accounted for in the final predictive model. Each axis represents a specific patient value, and the points awarded for each variable value were determined using the vertical lines drawn. Finally, the comprehensive score is presented on the total point axis, accompanied by a vertical line that extends downward to the survival axes to determine the probability of 5- and 3-year OS and PFS. In these two nomograms, the blue boxes represent the sample size of the demographic statistics, whereas the green block on the total point axis indicates the predicted population distribution of the prognosis (Data S1).

### Validation, evaluation, comparison, and risk stratification of nomograms

**Figure 4:**
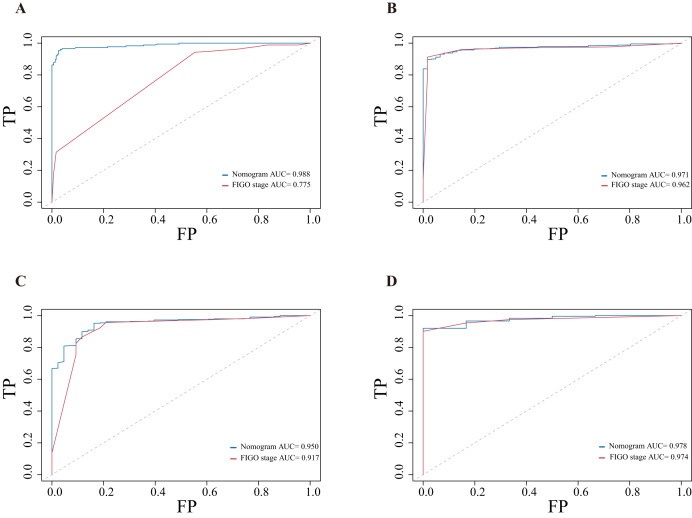
Receiver operating characteristic curves for nomograms and FIGO staging system. (A) Of 3-year overall survival; (B) of 5-year overall survival; (C) of 3-year progression-free survival; (D) of 5-year progression-free survival.

The nomograms were validated internally, and the ROC curves could be used to distinguish between patients who experienced an event and those who did not. Both nomograms used in our study had good distinguishing abilities (Fig. 4). At the same time, ROC curves were applied to compare the prognostic performance of the novel nomograms with that of the FIGO stage. The AUCs indicated that the predictive abilities of the nomograms were superior to those of the FIGO staging system (AUCs of 3-year OS, 0.988 *vs* 0.775; AUCs of 5-year OS, 0.971 *vs* 0.962; AUCs of 3-year PFS, 0.950 *vs* 0.917; AUCs of 5-year PFS, 0.978 *vs* 0.974).

**Figure 5:**
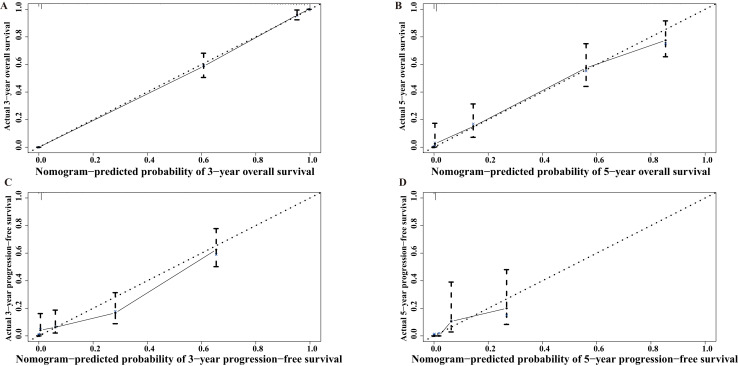
Calibration curves for nomograms. (A) Of 3-year overall survival; (B) of 5-year overall survival; (C) of 3-year progression-free survival; (D) of 5-year progression-free survival.

The calibration curves indicated excellent agreement between the predicted nomograms and actual survival outcomes (Fig. 5). The x-axis represents the nomogram-predicted probabilities of each patient’s 3-year OS, 5-year OS, 3-year PFS, and 5-year PFS, whereas the y-axis represents the actual 3-year OS, 5-year OS, 3-year PFS, and 5-year PFS for each patient. In general, if the solid line completely coincides with the dashed line, the model is ideal.

**Figure 6:**
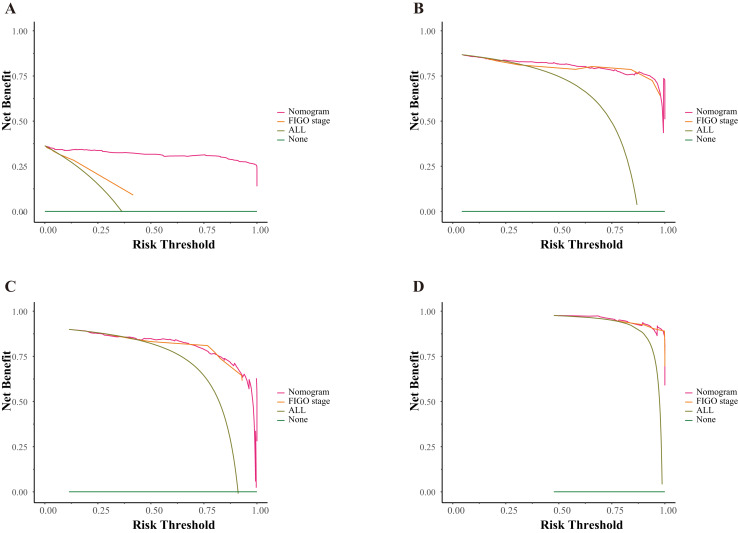
Decision curves for the nomograms and FIGO staging system. (A) Of 3-year overall survival; (B) of 5-year overall survival; (C) of 3-year progression-free survival; (D) of 5-year progression-free survival.

The DCA curves for the nomogram models are shown in Fig. 6. In the DCA curve, the x-axis indicates the threshold probability and the y-axis indicates the net benefit. The horizontal and oblique lines represent extreme situations when no patients or all patients underwent treatment, respectively. Typically, the curve with the greatest benefit represents the optimal treatment option, indicating high clinical utility. However, if the curves overlap, the optimal option is determined by the patient’s willingness to take risks. The curve for the 3-year OS prediction of the nomogram model was above the corresponding curve for the FIGO staging system, indicating that the net benefit of the former was superior to that of the latter. Although the DCA curves for the 5-year OS, 3-year PFS, and 5-year PFS intersected, the nomogram curves were approximately above the FIGO model curves, suggesting that the nomogram models provided favorable predictions. The DCA curves indicated that the nomograms have great potential for clinical applications.

**Figure 7:**
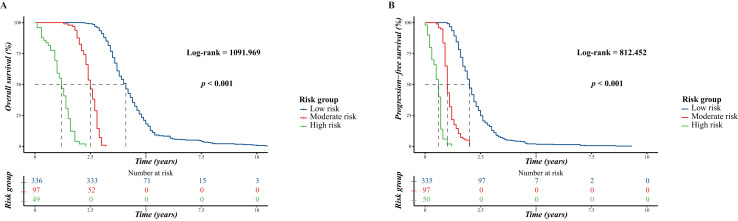
Kaplan-Meier curves of patients with high-grade serous ovarian carcinoma stratified by risk group. (A) For overall survival; (B) for progression-free survival.

We then calculated the total scores for all the patients based on the nomograms for risk stratification. Patients with HGSOC were classified into three risk groups based on the prediction of OS and PFS using the results from X-tile software. For OS, the low-risk scores ranged from 15.62 to 152.4, moderate-risk scores ranged from 153.51 to 206.14, and high-risk scores ranged from 209.19 to 277.99. For PFS, the low-risk scores ranged from 11.65 to 147.92, moderate-risk scores ranged from 148.55 to 226.25, and high-risk scores ranged from 226.99 to 296.87. According to the K–M survival curves shown in Fig. 7, statistical differences were observed among all three subgroups for the OS and PFS (*p* < 0.001), indicating the remarkable risk-stratification ability of our nomograms.

## Discussion

HGSOC is known as a “silent killer” because it is associated with no identifiable symptoms in its early stages. Although >60% of patients with OC achieve early remission, 70% of individuals with severe OC experience recurrence within 5 years of remission, and many develop resistance (Zhang et al., 2023). Further, survival and treatment response prediction are challenging but urgently required for patients with HGSOC. Thus, nomograms are useful risk quantification tools in clinical oncology. For example, Wang et al. (2021) collected limited data from a public database and developed two nomograms to predict OS and cancer-specific survival in patients with EOC. To assess prognosis and explore potential mechanisms of OC progression, Cong et al. (2024) developed a disulfidptosis-related prognostic signature and corresponding prognostic nomogram. However, nomograms integrating clinical characteristics for patients with HGSOC are lacking. Therefore, we included as many factors as possible in our study and developed two nomograms to predict OS and PFS in patients with HGSOC.

### Main findings and relevant literature

Here, the age at diagnosis was associated with OS and PFS in patients with HGSOC. With an increasing age, the OS and PFS of patients gradually decreased, with patients >66 years of age having the lowest OS and PFS rates. This could be related to the poor nutritional status of elderly patients, complex underlying diseases, and low tolerance to treatment (Cheng et al., 2023). Moreover, Pawelec (2019) proposed that older patients are more likely to have poor survival due to limited immune responses. As is generally accepted, a longer first-visit interval contributed to a poorer prognosis for HGSOC in our study. A longer first-visit interval was associated with a higher risk of tumor progression, which, in turn, was linked to a shorter OS and PFS. Balzano et al. (2022) reported that self-reported anxiety or depression is associated with longer diagnostic delays. Several studies have also shown that an inflammatory microenvironment can promote the occurrence and development of OC (Kandalaft, Dangaj Laniti & Coukos, 2022). Compared to individual blood parameters, the NLR is a superior inflammatory marker owing to its sensitivity and stability; moreover, it is significantly increased in OC and can be used to predict distant metastasis (Zhang et al., 2023). Similar to that in previous studies, patients with a higher NLR before the initial treatment had shorter OS and PFS rates in our study. Wang et al. (2016) reported that a high preoperative NLR has predictive value for both poor OS and PFS in patients with HGSOC, whereas Feng et al. (2018) found that a high NLR could predict poor PFS but not OS. However, Topcu et al. (2014) demonstrated that NLR is not an effective marker for predicting the malignant features of pelvic tumors. In our study, the cutoff values were ≤4.13, 4.13–6.11, and >6.11, which were similar to current common values, concentrated in the range of 0.89 to 6 (Zhang et al., 2023). Although some guidelines or consensuses emphasize the predictive value of NLR and they are still not widely used in clinical practice, Zhang et al. (2023) summarized their reliability in predicting OS and PFS in patients with HGSOC and concluded that NRL has great potential for predicting prognosis in these patients. Immunohistochemically, varying ER and PR expression levels have been linked to OS and PFS in patients with HGSOC. Our study showed that the ER (++) and PR (+++) status is associated with an improved OS and PFS in HGSOC. This can be explained by the fact that ER and PR mediate the effects of female steroid hormones on OC cell proliferation and apoptosis (Sieh et al., 2013). Furthermore, ER plays a role in regulating genome stability and influencing homologous recombination repair in HGSOC, whereas PR directly interacts with the breast cancer susceptibility gene (*BRCA1*), indicating platinum sensitivity (Tan et al., 2021). In this study, the prognosis of ER (+ + +) patients was worse than that of ER (+) patients, which differs from the results of Sieh et al. (2013). Additionally, our study found that patients with PR (++) had a shorter OS than those with PR (+++); however, this difference was not statistically significant in terms of the PFS. These two results could be due to the limited number of patients and the deletion of data. Traditionally, ER is expressed in more than 80% of HGSOC cases, whereas the PR-positivity rate in HGSOC ranges from 20% to 60% (Tan et al., 2021). Although there are numerous studies on prognostic biomarkers for HGSOC that have used immunohistochemical analysis, further research is needed to determine the prognostic and predictive values of ER and PR in HGSOC (Kok et al., 2019). In this study, more than 75% of the patients with HGSOC were diagnosed at advanced stages (FIGO stages III–IV), and a higher FIGO stage is indicative of lower OS and PFS rates. Moreover, patients with early-FIGO-stage disease have relatively small residual lesions, are sensitive to chemotherapy, and have a low risk of recurrence and metastasis, indicating a favorable prognosis (Liu et al., 2022). However, patients with late-FIGO-stage disease have tumor cells that migrate throughout their bodies, making complete surgical treatment challenging (Qi et al., 2022). Additionally, the drug resistance of advanced HGSOC is high and tolerance to chemotherapy is poor (Liu et al., 2022). Perhaps because of regional and missing data limitations, the relative risk of PFS in patients with stage IIC, IIIB, and IVB disease appeared to be relatively low, which has motivated us to pursue more extensive and comprehensive data-collection efforts in the future. In addition to the aforementioned variables, we found that patients with WT-1 (++) expression had a shorter OS than those with negative WT-1 expression. Typically, WT-1 is expressed in SOC and is considered a diagnostic marker (Hedley et al., 2014). Further, WT-1 controls cellular invasive activity, modulates cell migration in OC, and is regulated by the interstitial collagen microenvironment. Multiple studies on gynecological and non-gynecological cancers have assessed the predictive significance of WT-1 expression, yielding varied results. Angelico et al. (2020) observed a significant decline in OS with WT-1 expression, but McEachron et al. (2022) did not observe a difference in OS based on the WT-1 status. Moreover, NACT was found to have the ability to improve the OS of HGSOC, but it has several limitations and is primarily used to treat advanced diseases. Interval debulking surgery following NACT, which usually involves three cycles of chemotherapy, is an alternative treatment for patients who cannot undergo complete initial resection (Coleridge et al., 2021). Nikolaidi et al. (2022) found that NACT can be beneficial for HGSOC survival because it increases immune infiltration and programmed death ligand-1 (PDL-1) expression, induces local immune activation, and potentiates the immunogenicity of immune-excluded HGSOC. In terms of independent prognostic factors affecting PFS, we also found that patients with serum CA125 levels >5,000 U/mL had a longer PFS than those with serum CA125 levels <217.8 U/mL. The singular role of serum CA125 in predicting prognosis and platinum sensitivity remains debatable because of the associated low sensitivity and specificity (Jin et al., 2022). Wang et al. (2022) suggested that a high serum CA125 level before initial treatment is associated with poor survival outcomes in patients with EOC. Moreover, Asali et al. (2021) reported that patients with advanced HGSOC and low serum CA125 levels have the same clinical outcomes as those with higher levels, which can help to explain our results. Additionally, we found that the expression of CK7 was related to a short PFS in HGSOC. CK7 is less tissue-specific, and its expression has been observed in 59 of 85 epithelial tumor types (Fei et al., 2019). Dum et al. (2022) found that the overexpression of CK7 in HGSOC is associated with unfavorable tumor characteristics and a poor prognosis. Moreover, Ji et al. (2020) concluded that CK7 is linked to age, tissue differentiation, and the number of residual lesions in patients with OC and that positive CK7 expression is associated with postoperative recurrence in individuals with OC. In this study, patients with omentum metastasis had a poor PFS due to the advanced disease stages and limited treatment options associated with this condition (Zheng et al., 2023). We also found that postoperative adjuvant chemotherapy was associated with a longer PFS. It is recommended that patients with early-stage HGSOC undergo extensive staged surgery and postoperative chemotherapy with platinum-based drugs, whereas individuals with advanced HGSOC are advised to undergo cytoreductive surgery, followed by chemotherapy with paclitaxel and a platinum agent (Qi et al., 2022). Therefore, postoperative adjuvant chemotherapy could improve the prognosis of patients with HGSOC. Although the serum HE4 levels before treatment and R0 resection were not statistically significant predictive factors in our study, they play an important role in HGSOC prognosis. Notably, serum HE4 levels have a unique role in OC, as they are associated not only with an OC diagnosis but also with the prognosis and recurrence of this deadly neoplasm. Furthermore, achieving R0 CRS is commonly acknowledged as the most favorable prognostic factor for OC (Wang et al., 2019). In particular, current research suggests that every 10% R0 CRS enhances the survival rate of patients with OC by 5.5% (Bristow et al., 2002). Therefore, we included these variables in the development of the final two predictive models, based on their importance.

Since the remaining variables were not statistically significant in our study and existing studies could not determine their accurate prognostic significance, we did not include them in the final nomograms. However, their role in predicting HGSOC prognosis is worthy of attention. Some studies have suggested that increased body weight has a negative effect on HGSOC survival, whereas others did not identify this association (Chen et al., 2023). Of note, Kukla et al. (2022) found that adipose tissue can secrete adipokines that might influence the secretion of angiogenic factors, the growth and spread of neoplastic cells, and resistance to chemotherapy in various types of tumors. ABO blood groups might also influence the development of various types of cancers; however, the underlying mechanisms have not been thoroughly investigated (Gitas et al., 2020). At present, the reasons for the lack of a unified conclusion include differences in race, the influence of confounding factors, and the use of hospital-based control individuals (Song et al., 2019). The ovary is a heterogeneous endocrine organ containing an ovarian follicle. Some studies have found that risk factors for EOC include the number of lifetime ovulations, including the absence of pregnancy, an early age at menarche, and late age at menopause (Lheureux et al., 2019). To assess the effect of the ovulation time on HGSOC prognosis, we incorporated variables such as the age at menarche and menopause, as well as the number of pregnancies and miscarriages, for Cox regression analyses. Pretreatment CEA levels are independent prognostic factors for breast, colorectal, gastric, and lung cancers (Lin et al., 2020). However, strong evidence of their prognostic significance for patients with HGSOC is lacking. In 2018, Lin, Cao & Shen (2018) found that an elevated preoperative serum CEA level might indicate poor prognosis in OC, but Nomelini et al. (2017) reached the opposite conclusion. Moreover, RDW-CV and SII are associated with inflammation and are considered potential predictive markers of HGSOC; however, further studies are required to confirm this (Mao & Yang, 2023). Considering the role of inflammation in tumor progression and the convenience of hematological indicators, these could have great predictive potential in the future. Increasing attention has been paid to the clinical significance of the MCV and MCH in predicting the progression of tumors, including esophageal, colorectal, and liver cancers, in addition to their use in the diagnosis of hematological diseases (Wang & Zhang, 2022). Claps et al. (2022) reported that serum LDH, which is related to the prognosis of many tumors, is a complex biomarker associated with the activation of various oncogenic signaling pathways, metabolic activity, and immunogenicity in numerous tumors. Previous studies have also shown an association between serum lipids and tumor development and progression (Dai et al., 2016). In particular, actively proliferating tumor cells require a constant supply of lipids for membrane synthesis, whereas non-proliferating cells require sufficient lipids for enhanced signaling and resistance to apoptosis (Rysman et al., 2010). TG/free fatty acid cycling can activate nuclear factor kappa-B (NF-κB), which controls the expression of anti-apoptotic proteins through the G-protein coupled receptor (GPCR) pathway (Dai et al., 2016). Moreover, HDL-C is an essential component of host immunity and can suppress Toll-like receptor (TLR)-mediated tumor-promoting inflammation and levels of tumor survival signal promoters by activating the transcription factor activator of transcription factor 3 (ATF3) (De Nardo et al., 2014). Further prospective and multicenter studies are needed to enhance our understanding of the role of the TG/HDL-C ratio in HGSOC. The tumor size, laterality, and surgical modality can also affect the realization of R0 resection and the prognosis of patients with HGSOC. Further, the ascites microenvironment can promote tumor growth, chemotherapy resistance, and immune evasion, which could have crucial roles in HGSOC progression (Almeida-Nunes et al., 2023). Lymph node metastasis occurs in more than 45% of patients with advanced EOC and approximately 13% of patients with early-stage EOC and could be an independent risk factor for treatment resistance (Sun et al., 2023). However, most patients with advanced HGSOC exhibit ascites and lymph node metastases, and these factors might not be considered independent prognostic indicators for these patients. In our study, >60% of the patients had advanced-stage disease. This could explain why neither of these factors had prognostic significance in our study. Opinions regarding the correlation between immunohistochemical indicators and prognosis are complex and still divided (Atallah et al., 2023). Therefore, in a previous study, we included all relatively complete immunohistochemical indices detected at the Affiliated Hospital of Qingdao University.

The established nomograms were evaluated using a series of tests and were compared with the FIGO staging system. The validation results indicated good discriminatory performance and calibration, as well as the high clinical application value of our nomograms. The traditional FIGO staging system does not accurately assess the prognosis of HGSOC, as it only considers a limited number of essential prognostic markers. The predictive model developed by Xu et al. (2017) outperformed the current FIGO model. Similarly, our findings suggest that the nomogram could perform favorably, compared with the currently utilized FIGO staging system, for predicting OS and PFS in patients with HGSOC. Furthermore, our nomograms facilitated the risk categorization of patients with HGSOC, enabling more precise individualized prognostic stratification. By integrating multiple clinicopathological variables, the nomograms provide patient-specific estimates of OS and PFS, which may help identify different risk profiles within the same FIGO stage. This information can support individualized follow-up strategies, risk-adapted surveillance, and shared decision-making within current guideline-recommended treatment frameworks.

### Strengths and limitations

This study had several advantages. First, we developed nomograms using as many variables as possible, with the clinically important endpoints OS and PFS, indicating that our results are superior to those of previous studies. Second, the AUCs, calibration curves, and DCA curves demonstrated excellent predictive and discriminative performance, as well as the clinical utility of the models. Furthermore, the variables in these two nomograms can be easily collected and utilized in clinical practice. Third, compared to the FIGO staging system, our nomogram provided excellent clinical utility, and we established two new risk stratification systems for patients with HGSOC.

This study has several limitations. First, *BRCA* mutation and HRD status were not available for the included patients. As data were collected between 2008 and 2018, *BRCA* testing was not routinely implemented until after 2015, and HRD testing became common practice only after 2020. Given the important prognostic and therapeutic implications of these biomarkers, their absence represents a limitation of the current models. Second, chemotherapy response evaluated by RECIST criteria at 6 months was not consistently available and therefore could not be incorporated, which may have limited the ability to capture treatment efficacy in the prognostic analysis. Third, although the nomograms were internally validated, they are currently presented only in graphical form, and the lack of a web-based or software-based tool may limit immediate clinical applicability. Finally, socioeconomic factors were not included, as such data were not routinely collected, despite their potential influence on survival outcomes. Future studies incorporating molecular biomarkers, treatment response, comprehensive follow-up data, and socioeconomic variables, as well as implementing user-friendly digital tools, may further improve the robustness and clinical utility of prognostic models in HGSOC.

### Future directions

Our research indicates that in clinical practice, the management and prognosis of patients with HGSOC should be comprehensively assessed based on factors such as patient age, NLR, postoperative immunohistochemistry, and other relevant considerations. Simultaneously, utilizing methods such as nomograms to comprehensively evaluate the various clinical characteristics of patients is advantageous to ensure that they receive the most accurate and suitable treatment options available. However, a comprehensive predictive model to predict prognosis is lacking, which prompted this study. For example, although R0 is widely recognized as one of the strongest prognostic factors in ovarian cancer survival outcomes, in our cohort we observed that the K-M curves for R0 and R > 0 groups were closer than expected (Perrone et al., 2023). There are several possible explanations for this finding in our dataset. Firstly, the R > 0 group in our series includes a substantial proportion of patients who received effective adjuvant therapies and maintenance regimens, which may mitigate some of the disadvantage associated with residual disease. Secondly, variations in disease biology and patient characteristics (such as tumor burden, performance status, and comorbidities) may influence long-term outcomes independently of residual disease status. Thirdly, given the retrospective nature of the study and differences in surgical decision-making across the cohort, some patients classified as R > 0 had minimal residual tumor that may biologically behave more similarly to R0 resections. Lastly, although R0 remains a key prognostic determinant, statistical overlap between groups can occur when sample sizes in subgroups are limited or when confounding factors (such as treatment heterogeneity and follow-up duration) are present. Importantly, our multivariable analysis still showed the directional effect of resection status consistent with published data, and the similarity of survival curves does not diminish the established clinical importance of achieving R0 resection. These observations may reflect the specific characteristics of our cohort rather than contradict existing evidence. Future prospective studies with standardized surgical and treatment protocols are warranted to further clarify this relationship. We hope that this study will inspire the development of more comprehensive multicenter studies focusing on exploring predictive models that thoroughly assess the prognosis, diagnosis, and treatment of patients with HGSOC, to achieve precision medicine for these patients.

## Conclusions

Based on the data collected at the Affiliated Hospital of Qingdao University, we identified independent prognostic factors for patients with HGSOC. Furthermore, in our study, we developed and validated two nomograms to predict the 3- and 5-year OS and 3 and 5-year PFS of patients with HGSOC. Compared to the FIGO staging system, our predictive models showed strong predictive efficacy and significant clinical benefits. Additionally, two risk stratification systems were developed based on the risk scores generated from the nomograms. We hope that this research will encourage more in-depth studies that examine the comprehensive list of prognostic factors and validate our model using external datasets. This will assist doctors in conducting individualized diagnosis and treatment, ultimately enhancing the prognosis of patients with HGSOC.

## Supplemental Information

10.7717/peerj.21190/supp-1 Supplemental Information 1 Raw data.

10.7717/peerj.21190/supp-2 Supplemental Information 2 CodeBook.

10.7717/peerj.21190/supp-3 Supplemental Information 3 The example of nomograms.

10.7717/peerj.21190/supp-4 Supplemental Information 4 STROBE checklist.

10.7717/peerj.21190/supp-5 Supplemental Information 5 Kaplan-Meier curves for overall survival of patients with high-grade serous ovarian carcinoma. (A) Stratified by age of diagnosis; (B) stratified by BMI; (C) stratified by ABO blood group; (D) stratified by age at menarche; (E) stratified by age at menopause; (F) stratified by number of pregnancies; (G) stratified by number of miscarriages; (H) stratified by first visit interval; (I) stratified by serum CA125 level; (J) stratified by serum HE4 level; (K) stratified by serum CEA level; (L) stratified by RDW-CV; (M) stratified by MCV; (N) stratified by MCH; (O) stratified by NLR; (P) stratified by SII; (Q) stratified by LDH; (R) stratified by TG/HDL-C; (S) stratified by tumor size; (T) stratified by laterality; (U) stratified by the immunohistochemical expression of Ki-67; (V) stratified by the immunohistochemical expression of CA125; (W) stratified by the immunohistochemical expression of CK7; (X) stratified by the immunohistochemical expression of P16; (Y) stratified by the immunohistochemical expression of P53; (Z) stratified by the immunohistochemical expression of ER; (AA) stratified by the immunohistochemical expression of PR; (AB) stratified by the immunohistochemical expression of PAX-8; (AC) stratified by the immunohistochemical expression of Vimentin; (AD) stratified by the immunohistochemical expression of WT-1; (AE) stratified by surgical modality; (AF) stratified by R0 resection; (AG) stratified by ascites; (AH) stratified by lymph node metastasis; (AI) stratified by omentum metastasis; (AJ) stratified by FIGO stage; (AK) stratified by NACT; (AL) stratified by postoperative adjuvant chemotherapy.

10.7717/peerj.21190/supp-6 Supplemental Information 6 Kaplan-Meier curves for progression-free survival of patients with high-grade serous ovarian carcinoma. (A) Stratified by age of diagnosis; (B) stratified by BMI; (C) stratified by ABO blood group; (D) stratified by age at menarche; (E) stratified by age at menopause; (F) stratified by number of pregnancies; (G) stratified by number of miscarriages; (H) stratified by first visit interval; (I) stratified by serum CA125 level; (J) stratified by serum HE4 level; (K) stratified by serum CEA level; (L) stratified by RDW-CV; (M) stratified by MCV; (N) stratified by MCH; (O) stratified by NLR; (P) stratified by SII; (Q) stratified by LDH; (R) stratified by TG/HDL-C; (S) stratified by tumor size; (T) stratified by laterality; (U) stratified by the immunohistochemical expression of Ki-67; (V) stratified by the immunohistochemical expression of CA125; (W) stratified by the immunohistochemical expression of CK7; (X) stratified by the immunohistochemical expression of P16; (Y) stratified by the immunohistochemical expression of P53; (Z) stratified by the immunohistochemical expression of ER; (AA) stratified by the immunohistochemical expression of PR; (AB) stratified by the immunohistochemical expression of PAX-8; (AC) stratified by the immunohistochemical expression of Vimentin; (AD) stratified by the immunohistochemical expression of WT-1; (AE) stratified by surgical modality; (AF) stratified by R0 resection; (AG) stratified by ascites; (AH) stratified by lymph node metastasis; (AI) stratified by omentum metastasis; (AJ) stratified by FIGO stage; (AK) stratified by NACT; (AL) stratified by postoperative adjuvant chemotherapy.

## Additional Information and Declarations

### Data Availability

The following information was supplied regarding data availability:

The raw data is available in the Supplemental Files.
