# Supplementary material for: Construction of nomograms for predicting overall survival and progression-free survival in patients with high-grade serous ovarian carcinoma: a retrospective study
Source: PeerJ. 2026 Apr 30;14:e21190. doi: 10.7717/peerj.21190 (PMC13135751; doi:10.7717/peerj.21190)
Supplement: Supplemental Information 3 [file peerj-14-21190-s003.docx]

For example, a patient under the age of 59 years diagnosed with HGSOC, with a first-visit interval of 18–120 days, had serum CA125 and HE4 levels and an NLR of <217.8 U/mL, 668.24 pmol/L, and 4.13, respectively, before the initial treatment. She underwent R0 resection but did not undergo NACT or postoperative adjuvant chemotherapy, and her FIGO stage was IB. The immunohistochemical expression of ER, PR, and WT-1 was +++, ++, and +++, respectively. According to our nomogram for OS, the total number of points was 206, and the probabilities of an OS less than 3 and 5 years were 0.000448 and 0.162, respectively. Additionally, a patient having the same age at diagnosis, first-visit interval, serum CA125 and HE4 levels, NLR, immunohistochemical expression of ER, and FIGO stage did not show omental metastasis. Furthermore, the immunohistochemical expression of CK7 and PR was ++ and +++, respectively. She did not undergo R0 resection or NACT but received postoperative adjuvant chemotherapy. The total score for her was 234, and the probabilities of PFS being less than 3 and 5 years were 0.35 and 0.90, respectively, based on our nomogram.
