# Supplementary material for: Construction of nomograms for predicting overall survival and progression-free survival in patients with high-grade serous ovarian carcinoma: a retrospective study
Source: PeerJ. 2026 Apr 30;14:e21190. doi: 10.7717/peerj.21190 (PMC13135751; doi:10.7717/peerj.21190)
Supplement: Supplemental Information 4 [file peerj-14-21190-s004.docx]

STROBE Statement—checklist of items that should be included in reports of observational studies

|  | Item No. | Recommendation | Page  No. | Relevant text from manuscript |
| --- | --- | --- | --- | --- |
| **Title and abstract** | 1 | (*a*) Indicate the study’s design with a commonly used term in the title or the abstract | 2 | Information on patients primarily diagnosed with HGSOC at the Affiliated Hospital of Qingdao University from June 2008 to June 2018 was extracted. |
|  |  | (*b*) Provide in the abstract an informative and balanced summary of what was done and what was found | 2 | Methods. Information on patients primarily diagnosed with HGSOC at the Affiliated Hospital of Qingdao University from June 2008 to June 2018 was extracted. Kaplan–Meier (K-M) analyses were used to generate survival curves. Subsequently, we employed univariate and multivariate Cox regression analyses to determine independent prognostic factors, and prognostic nomograms for OS and PFS were developed based on these findings. We further compared the predictive models with the Federation of Gynecology and Obstetrics (FIGO) staging system and developed two new risk stratification systems.  Results. In total, 482 patients were included in the final study. The age at diagnosis, first-visit interval, peripheral blood neutrophil-to-lymphocyte ratio, the immunohistochemical expressions of estrogen receptor and progesterone receptor, and FIGO stage were independent prognostic factors associated with HGSOC OS and PFS. Additionally, the immunohistochemical expression of Wilms’ tumor-1 (WT-1) and neoadjuvant chemotherapy were also related to the OS, whereas the serum carbohydrate antigen 125 (CA125) level, the immunohistochemical expression of CK7, omentum metastasis, and postoperative adjuvant chemotherapy were independent prognostic factors linked to PFS. The area under the time-dependent receiver operating characteristic curve values of the nomograms were higher than those of the FIGO staging system, indicating good discrimination. The calibration curves and decision curve analysis curves were also well-calibrated and demonstrated the clinical applicability of the nomograms. We developed two new risk stratifications based on the total points of the nomograms. This study could provide a foundation for the development of more accurate predictive models that can assist clinicians in creating individualized treatment plans and improving the prognosis of HGSOC. |
| Introduction | | | |  |
| Background/rationale | 2 | Explain the scientific background and rationale for the investigation being reported | 3 | To date, only a few nomograms have been developed for patients with HGSOC, but most rely on relatively limited and variable clinical data and a comparison with the FIGO staging system, clinical benefit evaluation, and risk stratification are lacking |
| Objectives | 3 | State specific objectives, including any prespecified hypotheses | 3 | Therefore, this study was undertaken to establish and validate novel nomograms for overall survival (OS) and progression-free survival (PFS) in patients with HGSOC based on significant prognostic factors derived from the Affiliated Hospital of Qingdao University. We further compared methods for predicting prognosis using a nomogram and a separate FIGO staging system. Additionally, we used decision curve analysis (DCA) curves to assess the clinical utility of the nomograms and stratified the risk of HGSOC based on the nomograms. The goal of this study was to construct nomograms to predict the prognosis of patients with HGSOC and to promote the development of more comprehensive and reliable nomograms that can enhance individualized prognostic prediction and the treatment of HGSOC. |
| Methods | | | |  |
| Study design | 4 | Present key elements of study design early in the paper | 4 | The following variables were selected: age of diagnosis; body mass index (BMI); ABO blood group; age at menarche; age at menopause; number of pregnancies; number of miscarriages; first-visit interval; serum carbohydrate antigen 125 (CA125) level; serum human epididymis protein 4 (HE4) level; serum carcinoembryonic antigen (CEA) level; red blood cell distribution width-coefficient of variation (RDW-CV); mean corpuscular volume (MCV); mean corpuscular hemoglobin (MCH); peripheral blood neutrophil-to-lymphocyte ratio (NLR); systemic immunoinflammatory index (SII); lactate dehydrogenase (LDH); triglyceride-to-high density lipoprotein cholesterol ratio (TG/HDL-C); tumor size; tumor laterality; the immunohistochemical expression of Ki-67, CA125, CK7, P16, P53, estrogen receptor (ER), progesterone receptor (PR), PAX-8, vimentin, and Wilms’ tumor-1 (WT-1); surgical modality; R0 resection or not; ascites or not; lymph node metastasis or not; omentum metastasis or not; FIGO stage; neoadjuvant chemotherapy (NACT) or not; and postoperative adjuvant chemotherapy or not. In particular, the SII is an indicator of peripheral blood, calculated by multiplying the platelet count by the neutrophil count and then dividing by the lymphocyte count. Except for the immunohistochemical expression, FIGO stage, and treatment information, all other data were collected before the initial treatment, and peripheral blood indices were collected for the first time prior to the initial treatment. R0 resection was defined as no visible residual focus after R0 cytoreductive surgery (CRS) |
| Setting | 5 | Describe the setting, locations, and relevant dates, including periods of recruitment, exposure, follow-up, and data collection | 4 | All patients were followed up every 2–4 months in the first 2 years after completing primary treatment. The follow-up interval was then extended to every 3–6 months in the subsequent 3 years and further extended to every 6–12 months thereafter. The follow-up period was until June 2023. OS and PFS were the primary study endpoints; in addition, the 3-year OS, 5-year OS, 3-year PFS, and 5-year PFS were the other outcomes of interest in our study. OS was calculated from the date of diagnosis to the date of death from any cause, and PFS was calculated as the period from diagnosis to disease recurrence or progression. |
| Participants | 6 | (*a*) *Cohort study*—Give the eligibility criteria, and the sources and methods of selection of participants. Describe methods of follow-up  *Case-control study*—Give the eligibility criteria, and the sources and methods of case ascertainment and control selection. Give the rationale for the choice of cases and controls  *Cross-sectional study*—Give the eligibility criteria, and the sources and methods of selection of participants |  |  |
|  |  | (*b*) *Cohort study*—For matched studies, give matching criteria and number of exposed and unexposed  *Case-control study*—For matched studies, give matching criteria and the number of controls per case | 4 | The inclusion criteria were as follows: (1) initial treatment at the Affiliated Hospital of Qingdao University with either chemotherapy or surgery; (2) the patient underwent surgical treatment at the Affiliated Hospital of Qingdao University; (3) diagnosis of HGSOC by the Affiliated Hospital of Qingdao University after a postoperative pathological evaluation; and (4) complete clinical data. The exclusion criteria were as follows: (1) patients with other malignant tumors, such as cervical, breast, and gastric cancer; (2) patients with diseases that seriously affect survival, such as severe acute myocardial infarction and intracerebral hemorrhage; and (3) patients who were lost to follow-up. |
| Variables | 7 | Clearly define all outcomes, exposures, predictors, potential confounders, and effect modifiers. Give diagnostic criteria, if applicable | 4 | All patients were followed up every 2–4 months in the first 2 years after completing primary treatment. The follow-up interval was then extended to every 3–6 months in the subsequent 3 years and further extended to every 6–12 months thereafter. The follow-up period was until June 2023. OS and PFS were the primary study endpoints; in addition, the 3-year OS, 5-year OS, 3-year PFS, and 5-year PFS were the other outcomes of interest in our study. OS was calculated from the date of diagnosis to the date of death from any cause, and PFS was calculated as the period from diagnosis to disease recurrence or progression. |
| Data sources/ measurement | 8* | For each variable of interest, give sources of data and details of methods of assessment (measurement). Describe comparability of assessment methods if there is more than one group | 4 | In particular, the SII is an indicator of peripheral blood, calculated by multiplying the platelet count by the neutrophil count and then dividing by the lymphocyte count. Except for the immunohistochemical expression, FIGO stage, and treatment information, all other data were collected before the initial treatment, and peripheral blood indices were collected for the first time prior to the initial treatment. R0 resection was defined as no visible residual focus after R0 cytoreductive surgery (CRS) |
| Bias | 9 | Describe any efforts to address potential sources of bias | 4 | The exclusion criteria were as follows: (1) patients with other malignant tumors, such as cervical, breast, and gastric cancer; (2) patients with diseases that seriously affect survival, such as severe acute myocardial infarction and intracerebral hemorrhage; and (3) patients who were lost to follow-up. |
| Study size | 10 | Explain how the study size was arrived at | 5 | In total, 735 patients diagnosed with HGSOC were identified at the Affiliated Hospital of Qingdao University between June 2008 and June 2018. Of these, 482 were included based on the inclusion and exclusion criteria |

Continued on next page

| Quantitative variables | 11 | Explain how quantitative variables were handled in the analyses. If applicable, describe which groupings were chosen and why | 5 | X-tile software (Yale University, New Haven, Connecticut, USA) was used to convert continuous variables into categorical variables by calculating the optimal cutoff points for each variable. We then utilized Kaplan–Meier (K–M) survival analyses to calculate the survival probabilities, in terms of OS and PFS, of the clinical factors and created K–M curves. Finally, log-rank tests were used to compare the differences between the curves.  To assess several prognostic variables related to OS and PFS, univariate and multivariate Cox proportional hazard regression models were used to determine hazard ratios and 95% confidence intervals. Subsequently, two novel nomograms associated with OS and PFS were developed for patients with HGSOC based on the independent prognostic factors identified through multivariate analyses. |
| --- | --- | --- | --- | --- |
| Statistical methods | 12 | (*a*) Describe all statistical methods, including those used to control for confounding | 5 | All statistical analyses were performed using SPSS software (version 29.0; IBM Corp., USA) and R software (version 4.3.2; http://www.r-project.org/). A *p*-value <0.05 was considered statistically significant. |
|  |  | (*b*) Describe any methods used to examine subgroups and interactions | 5 | To assess the discriminatory ability of the nomograms, we used the area under the time-dependent receiver operating characteristic (ROC) curve (AUC). In addition, the performance of our nomogram was compared with that of the FIGO staging system using ROC curves. Calibration plots were created after 100 sampling repetitions, using the bootstrap method for internal validation. DCA curves were used to test the clinical applicability of the predictive models. Finally, two new risk stratification systems were developed using X-tile software based on the total points of the nomograms. Survival differences among the different risk stratification groups were compared using log-rank tests and K–M curves. |
|  |  | (*c*) Explain how missing data were addressed | 4 | The exclusion criteria were as follows: (1) patients with other malignant tumors, such as cervical, breast, and gastric cancer; (2) patients with diseases that seriously affect survival, such as severe acute myocardial infarction and intracerebral hemorrhage; and (3) patients who were lost to follow-up. |
|  |  | (*d*) *Cohort study*—If applicable, explain how loss to follow-up was addressed  *Case-control study*—If applicable, explain how matching of cases and controls was addressed  *Cross-sectional study*—If applicable, describe analytical methods taking account of sampling strategy | 4 | The exclusion criteria were as follows: (1) patients with other malignant tumors, such as cervical, breast, and gastric cancer; (2) patients with diseases that seriously affect survival, such as severe acute myocardial infarction and intracerebral hemorrhage; and (3) patients who were lost to follow-up. |
|  |  | (*e*) Describe any sensitivity analyses | 5 | To assess the discriminatory ability of the nomograms, we used the area under the time-dependent receiver operating characteristic (ROC) curve (AUC). In addition, the performance of our nomogram was compared with that of the FIGO staging system using ROC curves. Calibration plots were created after 100 sampling repetitions, using the bootstrap method for internal validation. DCA curves were used to test the clinical applicability of the predictive models. Finally, two new risk stratification systems were developed using X-tile software based on the total points of the nomograms. Survival differences among the different risk stratification groups were compared using log-rank tests and K–M curves. |
| Results | | | | |
| Participants | 13* | (a) Report numbers of individuals at each stage of study—eg numbers potentially eligible, examined for eligibility, confirmed eligible, included in the study, completing follow-up, and analysed | 5 | In total, 735 patients diagnosed with HGSOC were identified at the Affiliated Hospital of Qingdao University between June 2008 and June 2018. Of these, 482 were included based on the inclusion and exclusion criteria |
|  |  | (b) Give reasons for non-participation at each stage | 5 | The clinical characteristics of patients are presented in Table 1. Most cases were patients under 59 years of age (66.8%), and the highest proportion of first-visit intervals was 18–20 days (60.2%). The serum CA125 level for the majority of the patients was 217.8–2496 U/mL (61.6%), and their NLR was under 4.13 (69.7%). Although there were a considerable number of patients with omentum metastasis (61.2%) and stage IIIC disease (57.1%), most patients achieved R0 resection (82.6%) through general surgery (88.4%). Subsequently, the majority of patients underwent postoperative adjuvant chemotherapy (77.6%). The 3- and 5-year OS and PFS rates of all patients in terms of different clinical features are shown in Table 2. |
|  |  | (c) Consider use of a flow diagram | 5 | (Fig. 1) |
| Descriptive data | 14* | (a) Give characteristics of study participants (eg demographic, clinical, social) and information on exposures and potential confounders | 5 | The clinical characteristics of patients are presented in Table 1. Most cases were patients under 59 years of age (66.8%), and the highest proportion of first-visit intervals was 18–20 days (60.2%). The serum CA125 level for the majority of the patients was 217.8–2496 U/mL (61.6%), and their NLR was under 4.13 (69.7%). Although there were a considerable number of patients with omentum metastasis (61.2%) and stage IIIC disease (57.1%), most patients achieved R0 resection (82.6%) through general surgery (88.4%). Subsequently, the majority of patients underwent postoperative adjuvant chemotherapy (77.6%). The 3- and 5-year OS and PFS rates of all patients in terms of different clinical features are shown in Table 2. |
|  |  | (b) Indicate number of participants with missing data for each variable of interest | 5 | In total, 735 patients diagnosed with HGSOC were identified at the Affiliated Hospital of Qingdao University between June 2008 and June 2018. Of these, 482 were included based on the inclusion and exclusion criteria (Fig. 1). |
|  |  | (c) *Cohort study*—Summarise follow-up time (eg, average and total amount) | 6 | **Survival analyses and prognostic factors**  Fig. 2 and Fig. 3 display the K–M survival curves. According to K–M survival analyses, the age at diagnosis, BMI, first-visit interval, serum CA125 level, serum HE4 level, serum CEA level, NLR, SII, TG/HDL-C, immunohistochemical expression of Ki-67, CA125, P53, ER, PR, PAX-8, and WT-1, R0 resection or not, ascites or not, lymph node metastasis or not, omentum metastasis or not, NACT or not, postoperative adjuvant chemotherapy or not, and FIGO stage affected the OS and PFS of patients with HGSOC. Additionally, the LDH, tumor laterality, and immunohistochemical expression of P16 also affected the OS of patients with HGSOC. In addition, the immunohistochemical expression of CK7 affected patient PFS . Of note, crossed curves indicate potential interference from multiple sources.  Univariate and multivariate Cox proportional hazard regression analyses for OS and PFS are presented in Table 3 and Table 4, respectively. According to the results of univariate Cox regression analyses, the age at diagnosis, BMI, first-visit interval, serum CA125 level, serum HE4 level, NLR, SII, TG/HDL-C, immunohistochemical expressions of Ki-67, CA125, P53, ER, PR, PAX-8, and WT-1, ascites or not, lymph node metastasis or not, omentum metastasis or not, FIGO stage, NACT or not, and postoperative adjuvant chemotherapy or not were potentially correlated with the OS and PFS of patients with HGSOC. Besides, serum CEA level, LDH, tumor laterality, the immunohistochemical expression of P16, and R0 resection or not were risk factors for OS. Moreover, the immunohistochemical expression of CK7 was identified as a risk factor for PFS These potential prognostic factors were further assessed using multivariate Cox proportional hazard regression analyses, which indicated that the age at diagnosis, first-visit interval, NLR, ER, PR, WT-1, FIGO stage, and NACT were independent prognostic factors associated with the OS of HGSOC. Moreover, the age at diagnosis, first-visit interval, serum CA125 level, NLR, CK7, ER, PR, omentum metastasis, FIGO stage, and postoperative adjuvant chemotherapy were confirmed to be independent prognostic indicators of PFS. |
| Outcome data | 15* | *Cohort study—Report numbers of outcome events or summary measures over time* | 6 | **Survival analyses and prognostic factors**  Fig. 2 and Fig. 3 display the K–M survival curves. According to K–M survival analyses, the age at diagnosis, BMI, first-visit interval, serum CA125 level, serum HE4 level, serum CEA level, NLR, SII, TG/HDL-C, immunohistochemical expression of Ki-67, CA125, P53, ER, PR, PAX-8, and WT-1, R0 resection or not, ascites or not, lymph node metastasis or not, omentum metastasis or not, NACT or not, postoperative adjuvant chemotherapy or not, and FIGO stage affected the OS and PFS of patients with HGSOC. Additionally, the LDH, tumor laterality, and immunohistochemical expression of P16 also affected the OS of patients with HGSOC. In addition, the immunohistochemical expression of CK7 affected patient PFS . Of note, crossed curves indicate potential interference from multiple sources.  Univariate and multivariate Cox proportional hazard regression analyses for OS and PFS are presented in Table 3 and Table 4, respectively. According to the results of univariate Cox regression analyses, the age at diagnosis, BMI, first-visit interval, serum CA125 level, serum HE4 level, NLR, SII, TG/HDL-C, immunohistochemical expressions of Ki-67, CA125, P53, ER, PR, PAX-8, and WT-1, ascites or not, lymph node metastasis or not, omentum metastasis or not, FIGO stage, NACT or not, and postoperative adjuvant chemotherapy or not were potentially correlated with the OS and PFS of patients with HGSOC. Besides, serum CEA level, LDH, tumor laterality, the immunohistochemical expression of P16, and R0 resection or not were risk factors for OS. Moreover, the immunohistochemical expression of CK7 was identified as a risk factor for PFS These potential prognostic factors were further assessed using multivariate Cox proportional hazard regression analyses, which indicated that the age at diagnosis, first-visit interval, NLR, ER, PR, WT-1, FIGO stage, and NACT were independent prognostic factors associated with the OS of HGSOC. Moreover, the age at diagnosis, first-visit interval, serum CA125 level, NLR, CK7, ER, PR, omentum metastasis, FIGO stage, and postoperative adjuvant chemotherapy were confirmed to be independent prognostic indicators of PFS. |
|  |  | *Case-control study—*Report numbers in each exposure category, or summary measures of exposure |  |  |
|  |  | *Cross-sectional study—*Report numbers of outcome events or summary measures |  |  |
| Main results | 16 | (*a*) Give unadjusted estimates and, if applicable, confounder-adjusted estimates and their precision (eg, 95% confidence interval). Make clear which confounders were adjusted for and why they were included | 6-7 | Prognostic nomograms for predicting the OS and PFS at 3 and 5 years were constructed independently, based on the prognostic variables obtained (Fig. 4). In particular, we selected serum CA125 and HE4 levels, R0 resection, and postoperative adjuvant chemotherapy to construct a nomogram for OS, although these variables showed no significant differences in the multivariate Cox proportional hazard analyses of OS. Similarly, serum HE4 levels, R0 resection, and NACT were used to create a nomogram for the PFS of patients with HGSOC. Each axis represents a specific patient value, and the points awarded for each variable value were determined using the vertical lines drawn. Finally, the comprehensive score is presented on the total point axis, accompanied by a vertical line that extends downward to the survival axes to determine the probability of 5- and 3-year OS and PFS. In these two nomograms, the blue boxes represent the sample size of the demographic statistics, whereas the green block on the total point axis indicates the predicted population distribution of the prognosis.  For example, a patient under the age of 59 years diagnosed with HGSOC, with a first-visit interval of 18–120 days, had serum CA125 and HE4 levels and an NLR of <217.8 U/mL, 668.24 pmol/L, and 4.13, respectively, before the initial treatment. She underwent R0 resection but did not undergo NACT or postoperative adjuvant chemotherapy, and her FIGO stage was IB. The immunohistochemical expression of ER, PR, and WT-1 was +++, ++, and +++, respectively. According to our nomogram for OS, the total number of points was 206, and the probabilities of an OS less than 3 and 5 years were 0.000448 and 0.162, respectively. Additionally, a patient having the same age at diagnosis, first-visit interval, serum CA125 and HE4 levels, NLR, immunohistochemical expression of ER, and FIGO stage did not show omental metastasis. Furthermore, the immunohistochemical expression of CK7 and PR was ++ and +++, respectively. She did not undergo R0 resection or NACT but received postoperative adjuvant chemotherapy. The total score for her was 234, and the probabilities of PFS being less than 3 and 5 years were 0.35 and 0.90, respectively, based on our nomogram. |
|  |  | (*b*) Report category boundaries when continuous variables were categorized | 5 | X-tile software (Yale University, New Haven, Connecticut, USA) was used to convert continuous variables into categorical variables by calculating the optimal cutoff points for each variable. We then utilized Kaplan–Meier (K–M) survival analyses to calculate the survival probabilities, in terms of OS and PFS, of the clinical factors and created K–M curves. Finally, log-rank tests were used to compare the differences between the curves. |
|  |  | (*c*) If relevant, consider translating estimates of relative risk into absolute risk for a meaningful time period | 7 | The nomograms were validated internally, and the ROC curves could be used to distinguish between patients who experienced an event and those who did not. Generally, the AUC ranges from 0.5 to 1.0, with an AUC closer to 1 indicating that the predictive ability of the nomogram is more accurate. Typically, if the AUC is between 0.7 and 0.9, the model is considered to have a medium predictive value. If the AUC is >0.9, the model is considered to have good predictive value. Both nomograms used in our study had good distinguishing abilities (Fig. 5). At the same time, ROC curves were applied to compare the prognostic performance of the novel nomograms with that of the FIGO stage. The AUCs indicated that the predictive abilities of the nomograms were superior to those of the FIGO staging system (AUCs of 3-year OS, 0.988 *vs.* 0.775; AUCs of 5-year OS, 0.971 *vs.* 0.962; AUCs of 3-year PFS, 0.950 *vs.* 0.917; AUCs of 5-year PFS, 0.978 *vs.* 0.974). |

Continued on next page

| Other analyses | 17 | Report other analyses done—eg analyses of subgroups and interactions, and sensitivity analyses | 7-8 | The calibration curves indicated excellent agreement between the predicted nomograms and actual survival outcomes (Fig. 6). The x-axis represents the nomogram-predicted probabilities of each patient’s 3-year OS, 5-year OS, 3-year PFS, and 5-year PFS, whereas the y-axis represents the actual 3-year OS, 5-year OS, 3-year PFS, and 5-year PFS for each patient. In general, if the solid line completely coincides with the dashed line, the model is ideal.  The DCA curves for the nomogram models are shown in Fig. 7. In the DCA curve, the x-axis indicates the threshold probability and the y-axis indicates the net benefit. The horizontal and oblique lines represent extreme situations when no patients or all patients underwent treatment, respectively. Typically, the curve with the greatest benefit represents the optimal treatment option, indicating high clinical utility. However, if the curves overlap, the optimal option is determined by the patient’s willingness to take risks. The curve for the 3-year OS prediction of the nomogram model was above the corresponding curve for the FIGO staging system, indicating that the net benefit of the former was superior to that of the latter. Although the DCA curves for the 5-year OS, 3-year PFS, and 5-year PFS intersected, the nomogram curves were approximately above the FIGO model curves, suggesting that the nomogram models provided favorable predictions. The DCA curves indicated that the nomograms have great potential for clinical applications.  We then calculated the total scores for all the patients based on the nomograms for risk stratification. Patients with HGSOC were classified into three risk groups based on the prediction of OS and PFS using the results from X-tile software. For OS, the low-risk scores ranged from 15.62 to 152.4, moderate-risk scores ranged from 153.51 to 206.14, and high-risk scores ranged from 209.19 to 277.99. For PFS, the low-risk scores ranged from 11.65 to 147.92, moderate-risk scores ranged from 148.55 to 226.25, and high-risk scores ranged from 226.99 to 296.87. According to the K–M survival curves shown in Fig. 8, statistical differences were observed among all three subgroups for the OS and PFS (*p*<0.001), indicating the remarkable risk-stratification ability of our nomograms. |
| --- | --- | --- | --- | --- |
| Discussion | | | | |
| Key results | 18 | Summarise key results with reference to study objectives | 12 | This study had several advantages. First, we developed nomograms using as many variables as possible, with the clinically important endpoints OS and PFS, indicating that our results are superior to those of previous studies. Second, the AUCs, calibration curves, and DCA curves demonstrated excellent predictive and discriminative performance, as well as the clinical utility of the models. Furthermore, the variables in these two nomograms can be easily collected and utilized in clinical practice. Third, compared to the FIGO staging system, our nomogram provided excellent clinical utility, and we established two new risk stratification systems for patients with HGSOC. |
| Limitations | 19 | Discuss limitations of the study, taking into account sources of potential bias or imprecision. Discuss both direction and magnitude of any potential bias | 12 | However, it should be noted that there are several limitations to our study. First, the retrospective nature of the study might have led to selection bias. Second, detailed information about chemotherapy and other treatments was unavailable for this study. Third, the nomograms were not externally validated and might not be applicable to other regions. |
| Interpretation | 20 | Give a cautious overall interpretation of results considering objectives, limitations, multiplicity of analyses, results from similar studies, and other relevant evidence | 11-12 | The established nomograms were evaluated using a series of tests and were compared with the FIGO staging system. The validation results indicated good discriminatory performance and calibration, as well as the high clinical application value of our nomograms. The traditional FIGO staging system does not accurately assess the prognosis of HGSOC, as it only considers a limited number of essential prognostic markers. The predictive model developed by Xu et al. outperformed the current FIGO model (Xu et al. 2017). Similarly, our findings suggest that the nomogram could perform favorably, compared with the currently utilized FIGO staging system, for predicting OS and PFS in patients with HGSOC. Furthermore, our nomograms facilitated the risk categorization of patients with HGSOC, enabling more personalized treatment plans and follow-up schedules. |
| Generalisability | 21 | Discuss the generalisability (external validity) of the study results | 12 | HGSOC is known as a “silent killer” because it is associated with no identifiable symptoms in its early stages. Although >60% of patients with OC achieve early remission, 70% of individuals with severe OC experience recurrence within 5 years of remission, and many develop resistance (Zhang et al. 2023). Further, survival and treatment response prediction are challenging but urgently required for patients with HGSOC. Thus, nomograms are useful risk quantification tools in clinical oncology. For example, Wang et al. collected limited data from a public database and developed two nomograms to predict OS and cancer-specific survival in patients with EOC (Wang et al. 2021). To assess prognosis and explore potential mechanisms of OC progression, Cong et al. developed a disulfidptosis-related prognostic signature and corresponding prognostic nomogram (Cong et al. 2024). However, nomograms integrating clinical characteristics for patients with HGSOC are lacking. Therefore, we included as many factors as possible in our study and developed two nomograms to predict OS and PFS in patients with HGSOC. Our research indicates that in clinical practice, the management and prognosis of patients with HGSOC should be comprehensively assessed based on factors such as patient age, NLR, postoperative immunohistochemistry, and other relevant considerations. Simultaneously, utilizing methods such as nomograms to comprehensively evaluate the various clinical characteristics of patients is advantageous to ensure that they receive the most accurate and suitable treatment options available. However, a comprehensive predictive model to predict prognosis is lacking, which prompted this study. We hope that this study will inspire the development of more comprehensive multicenter studies focusing on exploring predictive models that thoroughly assess the prognosis, diagnosis, and treatment of patients with HGSOC, to achieve precision medicine for these patients. |
| Other information | |  | | |
| Funding | 22 | Give the source of funding and the role of the funders for the present study and, if applicable, for the original study on which the present article is based | Not applicable | Not applicable |

*Give information separately for cases and controls in case-control studies and, if applicable, for exposed and unexposed groups in cohort and cross-sectional studies.

**Note:** An Explanation and Elaboration article discusses each checklist item and gives methodological background and published examples of transparent reporting. The STROBE checklist is best used in conjunction with this article (freely available on the Web sites of PLoS Medicine at http://www.plosmedicine.org/, Annals of Internal Medicine at http://www.annals.org/, and Epidemiology at http://www.epidem.com/). Information on the STROBE Initiative is available at www.strobe-statement.org.
